# The Relationship Between a Low‐Carbohydrate Diet and the Prevalence of Diabetic Kidney Disease in Patients With Type 2 Diabetes: A Cross‐Sectional Study

**DOI:** 10.1002/fsn3.71462

**Published:** 2026-03-19

**Authors:** Wuyu Gao, Rong Ma, Hui Gu, Xue Zhang, Hao Liu, Mao Xiao, Zhuoxing Li, Yiting Wang, Yun Sun, Xiang Xiao

**Affiliations:** ^1^ Department of Nephrology People's Hospital of Xindu District Chengdu China; ^2^ Department of Clinical Medicine Chengdu Medical College Chengdu China; ^3^ People's Hospital of Xindu District Chengdu China; ^4^ Department of Endocrinology and Metabolism, The First People's Hospital of Yunnan Province The Affiliated Hospital of Kunming University of Science and Technology Kunming China

**Keywords:** DKD, low‐carbohydrate dietary, low‐carbohydrate dietary score, NHANES, risk factor, T2DM

## Abstract

Diabetic kidney disease (DKD) affects over 40% of patients with type 2 diabetes mellitus (T2DM), yet effective preventive strategies remain limited. Although low‐carbohydrate dietary (LCD) shows promise in glycemic control, their association with DKD prevalence is poorly understood. This study aimed to investigate the relationship between LCD adherence and DKD risk in a nationally representative T2DM population. 4558 adults with T2DM from the U.S. National Health and Nutrition Examination Survey were included (NHANES, 2009–2018). LCD adherence was quantified using the validated Low‐Carbohydrate Diet Score (LCDS, range 0–30), calculated from two 24‐h dietary recalls. DKD was defined as albumin‐to‐creatinine ratio ≥ 30 mg/g or estimated glomerular filtration rate < 60 mL/min/1.73 m^2^. Restricted cubic splines (RCS) modeled non‐linear associations, and weighted multivariable logistic regression analysis was conducted to explore the relationship between LCDS and the prevalence of DKD in T2DM patients, along with stratified analyses to assess the interaction of the results. The mean LCDS was 12.058, with higher scores in males, high‐income individuals, and insulin users (all *p* < 0.05). RCS revealed a linear inverse association between LCDS and DKD prevalence (*p* for nonlinearity = 0.490). In fully adjusted models, participants in the highest LCDS tertile (T3 vs. T1) had 21.5% lower DKD prevalence (OR = 0.785; 95% CI, 0.628–0.982; *p* = 0.034). Stratified analyses demonstrated no significant interaction effects between LCDS and covariates including age, sex, body mass index, or HbA1c (all *p* for interaction > 0.05). These findings highlight that higher LCDS was inversely associated with DKD prevalence in patients with T2DM. Future prospective studies should validate causality and optimize LCD protocols for renal protection.

## Graphical abstract

Based on a nationally representative study of individuals with type 2 diabetes mellitus (T2DM), a linear inverse association was observed between the Low‐Carbohydrate Diet Score (LCDS) and the prevalence of diabetic kidney disease (DKD). Individuals in the highest tertile of LCDS exhibited a significantly lower risk of DKD compared to those in the lowest tertile. This association remained consistent across various subgroups, including those stratified by age, sex, body mass index, and glycated hemoglobin (HbA1c) levels.

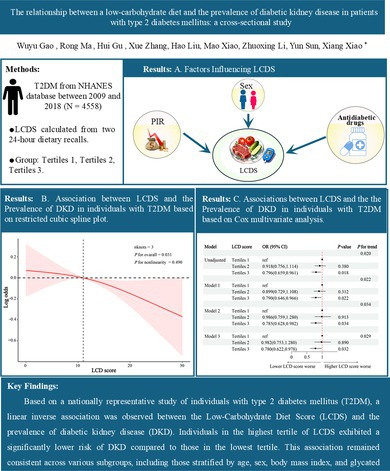

## 1 Introduction

Diabetes represents a widespread and serious health concern with significant global impact. There were 529 million people with diabetes worldwide across all age groups, with a prevalence rate of 6.1%, making diabetes one of the top ten causes of death and disability (Ma et al. 2025). In the United States, over 40% of type 2 diabetes mellitus (T2DM) patients developed diabetic kidney disease (DKD; Doshi and Friedman 2017). As one of the most prevalent microvascular complications of diabetes, DKD has become the primary cause of end‐stage renal disease (ESRD), imposing substantial clinical and economic burdens on both patients and society (Bakris et al. 2020).

Medical nutrition therapy (MNT) serves a pivotal role in DKD management. The development of effective nutritional strategies for preventing DKD progression is therefore critically important. Low‐carbohydrate dietary (LCD) patterns, including very‐low‐carbohydrate or ketogenic diets, are characterized by restricted daily carbohydrate intake with increased proportions of protein and fat consumption (Athinarayanan et al. 2024). Growing evidence supports their benefits for weight reduction, glycemic control, and lipid management in diabetic populations (Dyson et al. 2011; Wheeler et al. 2012). A systematic review and meta‐analysis concluded that six‐month adherence to a LCD may induce diabetes remission without clinically significant adverse events in patients with type 2 diabetes (Goldenberg et al. 2021).

Nevertheless, potential risks associated with LCDs warrant careful consideration. However, the relatively higher protein intake inherent to LCD raises concerns regarding potential renal impairment (Friedman 2004). Commonly reported gastrointestinal disturbances such as nausea, vomiting, constipation, diarrhea, and fat intolerance may also limit tolerability in certain patients (Klein et al. 2010; Zhang and Yang 2016). Additionally, elevated ketone body production may also lead to dehydration and electrolyte loss through frequent urinary excretion (Kang et al. 2004). Although LCD has been widely studied in blood glucose management, its long‐term effects on DKD remain inconclusive.

Several LCD variants have been proposed, including the Atkins, Zone, South Beach, and Paleo diets (Brouns 2018). The term LCD encompasses multiple nutritional regimens without a standardized definition, and clinical studies often lack detailed information about carbohydrate quantity and quality. To address this limitation, the low‐carbohydrate‐diet score (LCDS) was developed as a quantitative measure of dietary adherence (Sangsefidi et al. 2021). The LCDS was calculated according to methods established in prior nutritional epidemiology studies (Hu et al. 2023; Cao et al. 2025), which quantify adherence to LCD patterns by assigning weighted scores to macronutrient intake. This scoring system's validity lies in its ability to reflect the relative contributions of carbohydrates, proteins, and fats to total energy intake, thereby capturing the characteristic composition of low‐carbohydrate interventions (Ludwig et al. 2018). In recent years, the LCDS scoring system has been widely employed in large‐scale studies (Hu et al. 2023; Cao et al. 2025). Among diabetic populations, LCDS demonstrates strong correlations with biomarkers of glycemic control (reduced HbA1c, β‐hydroxybutyrate levels) and renal function (notably, urine albumin‐to‐creatinine ratio), confirming its construct validity (Hu et al. 2023).

In summary, although LCD have been extensively studied for glycemic management and weight loss, the potential relationship between LCD and the prevalence of DKD remains unclear. Therefore, this study hypothesizes that patients with a higher LCDS have a lower prevalence of DKD.

## 2 Materials and Methods

In this cross‐sectional study, all participant data were obtained from the National Health and Nutrition Examination Survey (NHANES) database (2009–2018). NHANES was a research program designed to assess the health and nutritional status of individuals in the United States (Ezzati et al. 1992). The survey employed a stratified, multistage probability sampling design to obtain nationally representative samples of the US civilian population, with data collection occurring biennially. We included participants diagnosed with T2DM between 2009 and 2018. The NHANES protocol included demographic, socioeconomic, dietary, and health‐related questionnaires during interviews, while the examination component comprised medical, dental, and physiological measurements along with laboratory tests conducted by trained medical personnel. These data were used to determine prevalence rates of major diseases and their associated prevalence factors, providing valuable information for evaluating relationships between nutritional status and health promotion/disease prevention.

### 2.1 Data Collection

Demographic and clinical parameters were systematically extracted from five consecutive NHANES survey cycles (2009–2018), encompassing: Ma et al. (2025) baseline characteristics: age, sex, race, and socioeconomic indicators (educational attainment, poverty‐income ratio, PIR; Doshi and Friedman 2017) metabolic profiles: body mass index, HbA1c levels, lipid panel components (Bakris et al. 2020) renal function markers: estimated glomerular filtration rate (calculated using the CKD‐EPI creatinine equation) and urinary albumin‐to‐creatinine ratio (Athinarayanan et al. 2024) behavioral factors: smoking status (current/former/never), alcohol consumption patterns (Dyson et al. 2011) comorbidity status: physician‐diagnosed hypertension and dyslipidemia (Wheeler et al. 2012) glucose‐lowering therapies: insulin usage and oral antidiabetic agents. The calculation of BMI was performed by dividing weight by height squared. Operational definitions for hypertension, dyslipidemia, smoking, and alcohol use were provided in Table S1.

### 2.2 Dietary Assessment

All NHANES participants were required to complete an initial 24‐h dietary recall interview at mobile examination centers, followed by a second telephone interview 3–10 days later. For participants in the NHANES database, dietary intake data were used to estimate the types and quantities of foods and beverages consumed during the 24‐h period preceding each interview (midnight to midnight), along with assessments of energy, nutrient, and food component content. In this study, we used the average of two 24‐h dietary recalls to estimate habitual dietary intake.

### 2.3 LCDS Calculation

The LCDS was calculated based on the methodology established in prior nutritional epidemiology studies (Hu et al. 2023; Cao et al. 2025), which quantifies adherence to a LCD pattern by assigning weighted scores to macronutrient intake. The rationale for this scoring system lies in its ability to reflect the relative contribution of carbohydrates, protein, and fat to total energy intake, thereby capturing the dietary composition typical of low‐carbohydrate interventions (Ludwig et al. 2018). Specifically, the LCDS scoring system was widely used in large‐scale studies (Hu et al. 2023; Cao et al. 2025). In diabetic populations, the LCDS demonstrated strong correlations with biomarkers of glycemic control (such as HbA1c reduction, β‐hydroxybutyrate levels) and renal function (e.g., urine albumin‐to‐creatinine ratio), confirming its construct validity (Hu et al. 2023). In this study, the LCDS was calculated as follows: Ma et al. (2025) Carbohydrate, protein, and fat intake (g) were converted to kilocalories using conversion factors of 4 kcal/g for carbohydrates and protein, and 9 kcal/g for fat (Doshi and Friedman 2017). The percentage of total energy derived from each macronutrient was calculated (Bakris et al. 2020). Scores were assigned based on tertiles of energy contribution: for carbohydrates, the lowest tertile (i.e., lowest carbohydrate intake) received 10 points, decreasing linearly to 0 points for the highest tertile; conversely, for fat and protein, the highest tertile received 10 points (Athinarayanan et al. 2024). The total LCDS ranged from 0 (lowest adherence) to 30 (highest adherence). Specific scoring criteria could be found in Table S2. In this study, the LCDS was categorized using the tertile method (Tertiles 1: < 7, Tertiles 2: 7–15, Tertiles 3: ≥ 15).

### 2.4 Diagnostic Criteria for Diabetes and DKD

To ensure diagnostic accuracy, we applied specific criteria for diabetes and DKD. Diabetes diagnosis required: (1) physician diagnosis, (2) HbA1c > 6.5%, (3) fasting plasma glucose ≥ 7.0 mmol/L, (4) random plasma glucose ≥ 11.1 mmol/L, or (5) 2‐h post‐OGTT plasma glucose ≥ 11.1 mmol/L, (6) use of diabetes medication or insulin; the diagnosis of diabetes required meeting at least one of the six. DKD diagnosis was based on diabetes diagnosis plus chronic kidney disease (CKD) criteria aligned with KDIGO guidelines: ACR > 30 mg/g or eGFR < 60 mL/min/1.73 m^2^ (Ludwig et al. 2018). Exclusion criteria included: (1) age < 20 years, (2) pregnancy, (3) non‐diabetic status, (4) type 1 diabetes (Patients with type 1 diabetes were excluded to ensure the homogeneity of the study population, that is, to focus exclusively on individuals with type 2 diabetes, and to avoid potential confounding bias arising from the fundamental differences in etiology and pathophysiology between these two distinct forms of diabetes), (5) absence of CKD diagnosis, and (6) missing LCDS data. Baseline was defined as the time of NHANES study entry. Rigorous laboratory testing included baseline ACR and eGFR measurements as detailed in the NHANES Laboratory Procedures Manual (Yuan et al. 2024). The study protocol complied with ethical standards of the 1964 Declaration of Helsinki and its later amendments, with approval from the NCHS Research Ethics Review Board. All participants provided written informed consent (Ma et al. 2023).

### 2.5 Statistical Analysis

Analyses followed CDC guidelines for complex survey weighting using NHANES sample weights (Chen et al. 2020). We calculated weights based on included variables to determine the minimal sufficient set, selected appropriate weights, and combined weights across survey years. The stratified, clustered survey design was accounted for to produce nationally representative estimates. Missing data were handled using multiple imputation. The imputation procedure was conducted with 5 replications (*m* = 5) and a maximum of 20 iterations per dataset. The specific algorithms applied were predictive mean matching (PMM) for continuous variables, logistic regression (logreg) for binary variables, polytomous logistic regression (polyreg) for unordered categorical variables, and proportional odds logistic regression (polr) for ordered categorical variables. Following NHANES analytical guidelines, continuous variables were summarized as weighted means (standard errors, SE) and categorical variables as percentages (SE). Weighted *t*‐tests or ANOVA were used for continuous variables, while weighted chi‐square tests assessed between‐group differences for categorical variables. Weighted multivariable linear regression models examined clinical predictors of LCDS in T2DM patients. Adjustment variables were selected based on their established roles in DKD pathogenesis from prior epidemiological studies (Afkarian et al. 2016; Alicic et al. 2017), including age (“< 60 years” or “≥ 60 years”), sex (“male” or “female”), BMI (“< 30 kg/m^2^” or “≥ 30 kg/m^2^”), hypertension (“Yes” or “No”), hyperlipidemia (“Yes” or “No”), and glycated hemoglobin levels (“< 7.0” or “≥ 7.0”). Adjust for age, sex (“Female,” “Male”), race (“Mexican American,” “Non‐Hispanic Black,” “Non‐Hispanic White,” “Other Hispanic,” “Other Race—Including Multi‐Racial”), BMI, smoking (“Yes” or “No”), alcohol use (“Yes” or “No”), education (“College or above,” “High school or equivalent,” “Less than high school”), PIR (“0–1,” “1.1–3,” “> 3”), HbA1c, antidiabetic drugs (“None,” “OHAS,” “Insulin,” “OHAS + Insulin”), hyperlipidemia (“Yes” or “No”), hypertension (“Yes” or “No”). Trend *p*‐values across LCDS tertiles were calculated using median values of each tertile as continuous variables, reflecting dose–response relationships without requiring multiple comparison adjustments. Weighted restricted cubic splines (RCS) evaluated dose–response relationships between LCDS and DKD prevalence. Multicollinearity was evaluated using variance inflation factors (VIF), with all covariates demonstrating VIF < 5, consistent with accepted thresholds (Hair et al. 2013). In sensitivity analyses, we conducted stratified analyses to ensure the robustness of our findings. Additionally, to minimize the impact of missing data, we excluded cases with missing values and repeated the analyses. Two‐tailed *p*‐values < 0.05 were considered statistically significant. All statistical analyses were performed using R 4.2.2.

## 3 Results

### 3.1 Baseline Characteristics

**FIGURE 1:**
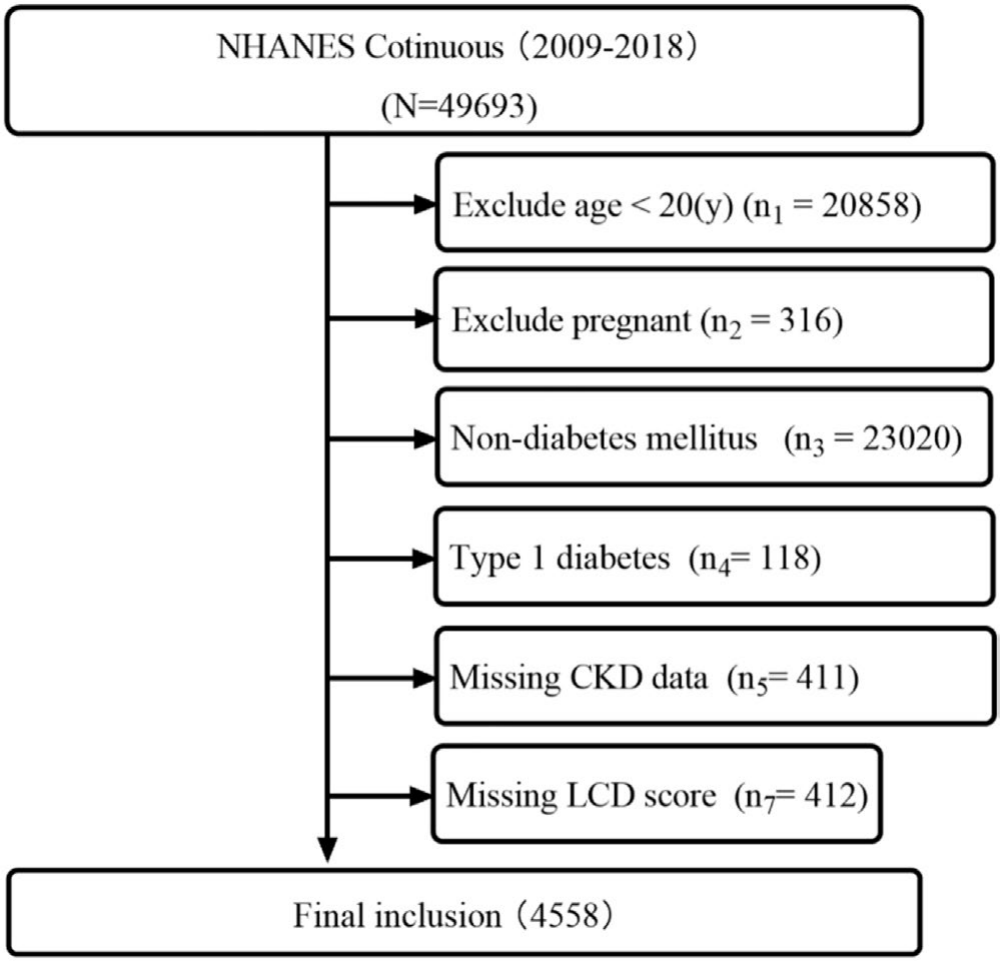
Flowchart of study enrollment.

From 2009 to 2018, data from 49,693 individuals were recorded in the NHANES database (Figure 1). The analysis included a total of 4558 observations. Among these, 3097 (67.9%) were complete cases, resulting in a missing data proportion of 32.1%. Table S3 presents the baseline clinical features of enrolled individuals with T2D, before and after multiple imputation. Missing values were identified in 10 variables, with rates ranging from 2.2% to 18.7%. The variable “Antidiabetic drugs” exhibited the highest proportion of missing values (18.7%; Figure S1). Convergence diagnostics indicated satisfactory convergence for all imputed variables (Figure S2). After screening, 4558 individuals who met the inclusion–exclusion criteria were included in the study, of which 48.801% were female, with an average age of 59.872 years. The prevalence of comorbidity with hypertension was 70.994%, and hyperlipidemia was 88.520%, with an average HbA1c level of 7.073 (Figure 1, Table 1).

**TABLE 1:** Baseline characteristics of the study participants with T2D.

Variable	Total (*N* = 4558)	T1 (0 ≤ LCDS ≤ 7; *n* = 1570)	T2 (7 < LCDS ≤ 15; *n* = 1544)	T3 (15 ≤ LCDS < 30; *n* = 1444)	*p*
LCDS	12.058 (0.152)	3.106 (0.081)	11.492 (0.076)	20.872 (0.166)	< 0.001
Age (years)	59.872 (0.328)	59.676 (0.553)	60.094 (0.444)	59.823 (0.394)	0.784
Sex (%)					0.009
Female	48.801 (0.019)	53.479 (2.337)	50.110 (2.349)	43.153 (1.895)	
Male	51.199 (0.018)	46.521 (2.337)	49.890 (2.349)	56.847 (1.895)	
Race (%)					< 0.001
Mexican American	10.350 (0.013)	12.373 (1.758)	9.292 (1.223)	9.575 (1.406)	
Non‐Hispanic Black	13.756 (0.010)	14.865 (1.290)	12.849 (1.457)	13.669 (1.349)	
Non‐Hispanic White	60.070 (0.031)	52.861 (2.356)	63.957 (2.205)	62.704 (2.312)	
Other Hispanic	6.413 (0.006)	8.243 (0.960)	6.237 (0.830)	4.912 (0.727)	
Other race—including multi‐racial	9.411 (0.007)	11.658 (1.055)	7.665 (0.779)	9.140 (1.112)	
PIR (10,000 dollars), %					< 0.001
> 3	38.874 (0.017)	34.406 (2.014)	42.300 (2.216)	48.260 (2.202)	
1.1–3	15.630 (0.009)	20.346 (1.776)	17.650 (1.314)	12.916 (1.149)	
0–1	38.236 (0.017)	45.248 (1.733)	40.051 (1.958)	38.824 (2.023)	
Educational level (%)					0.002
College or above	52.445 (0.019)	47.477 (2.559)	55.832 (1.859)	53.655 (2.286)	
High school or equivalent	37.932 (0.018)	39.269 (2.637)	35.181 (1.642)	39.615 (2.429)	
Less than high school	9.545 (0.008)	13.254 (1.417)	8.987 (0.911)	6.730 (0.818)	
BMI (%)					0.813
Underweight	0.265 (0.001)	0.241 (0.130)	0.190 (0.086)	0.373 (0.196)	
Normal weight	10.785 (0.007)	11.689 (1.013)	10.119 (1.105)	11.026 (1.293)	
Overweight	24.995 (0.010)	25.842 (1.318)	25.868 (1.376)	24.232 (1.549)	
Obesity	62.742 (0.023)	62.227 (1.659)	63.823 (1.678)	64.369 (2.037)	
Alcohol use (%)					0.006
No	13.335 (0.008)	16.742 (1.371)	16.269 (1.530)	10.868 (1.214)	
Yes	77.931 (0.026)	83.258 (1.371)	83.731 (1.530)	89.132 (1.214)	
Smoke (%)					0.095
No	50.207 (0.014)	50.863 (1.978)	52.769 (1.923)	47.011 (1.869)	
Yes	49.767 (0.021)	49.137 (1.978)	47.231 (1.923)	52.989 (1.869)	
e‐GFR (ml/min/1.73m^2^)	83.491 (0.538)	83.254 (0.872)	83.141 (0.857)	84.069 (0.811)	0.650
e‐GFR (mL/min/1.73m^2^), %					0.238
≥ 60	81.242 (0.026)	81.343 (1.263)	80.890 (1.390)	83.930 (1.542)	
< 60	17.760 (0.010)	18.657 (1.263)	19.110 (1.390)	16.070 (1.542)	
ACR (mg/g)	108.671 (8.897)	108.043 (12.485)	106.668 (14.144)	111.302 (17.719)	0.979
ACR (mg/g), %					0.783
< 30	73.312 (0.023)	72.359 (1.542)	74.218 (1.735)	75.062 (1.748)	
30–60	19.706 (0.011)	21.277 (1.384)	19.286 (1.569)	19.179 (1.552)	
≥ 300	6.153 (0.006)	6.364 (0.913)	6.495 (1.039)	5.759 (0.803)	
HbA1c (%)	7.073 (0.033)	7.066 (0.054)	7.001 (0.056)	7.155 (0.072)	0.313
Hyperlipidemia (%)					0.698
No	11.472 (0.007)	12.105 (1.221)	10.846 (0.969)	11.536 (1.060)	
Yes	88.520 (0.027)	87.895 (1.221)	89.154 (0.969)	88.464 (1.060)	
Hypertension (%)					0.200
No	29.006 (0.015)	31.774 (1.761)	28.149 (2.041)	27.342 (1.829)	
Yes	70.994 (0.022)	68.226 (1.761)	71.851 (2.041)	72.658 (1.829)	
Antidiabetic drugs (%)					0.471
None	21.405 (0.012)	27.178 (1.769)	26.630 (1.848)	24.635 (1.932)	
OHAS	43.463 (0.015)	53.173 (1.838)	53.725 (2.062)	52.065 (2.166)	
Insulin	6.239 (0.005)	6.251 (0.848)	7.555 (1.081)	8.800 (1.021)	
OHAS + Insulin	10.950 (0.007)	13.398 (1.243)	12.091 (1.078)	14.500 (1.324)	
Energy intake (kca/day)	1978.326 (24.952)	1834.568 (34.590)	2023.328 (47.978)	2061.610 (38.928)	< 0.001

Abbreviations: ACR, albumin‐creatinine ratio; BMI, body mass index; e‐GFR, estimated glomerular filtration rate; LCDS, low‐carbohydrate‐diet score; OHAS, Oral hypoglycaemic agents; PIR, poverty‐income ratio.

The average LCDS for T2DM patients was 12.058 in the United States. Among the included population, there were 34.444% (1570/4558) people in Tertiles 1, the average LCDS was 3.106; 33.875% (1544/4558) people in Tertiles 2, the average LCDS was 11.492; 31.681% (1444/4558) in Tertiles 3, the average LCDS was 20.872. In addition, in the population with a high LCDS, the proportion of males was relatively high, the proportion of non‐Hispanic whites was relatively high, and there was no significant age difference (Table 1). Over time, the proportion of T2DM patients with higher LDCS has been increasing annually (Table S4).

Meanwhile, significant differences were observed in indicators such as alcohol consumption, education, and PIR among the different groups (all *p* < 0.05). However, the proportion of hypertension and hyperlipidemia among patients in different groups, along with the levels of HbA1c, eGFR, and ACR, demonstrated no significant differences (all *p* > 0.05, Table 1).

### 3.2 Potential Determinants of LCDS

A multiple linear regression model was employed to analyze the impact of clinical indicators on the dietary choices of T2DM patients concerning LCD. After multivariable adjustment, the results indicated that factors such as sex, PIR, and type of antidiabetic drugs significantly influenced LCDS among T2DM patients (all *p* < 0.05, Table 2).

**TABLE 2:** Association between Clinical Characteristics and the LCDS in Patients with T2D: A Linear Regression Analysis.

Variable	*β*	95% CI	*p*
Age	0.003	(−0.043, 0.049)	0.886
Sex			
Female	Ref	Ref	Ref
Male	1.241	(0.402, 2.080)	0.004
Race			
Mexican American	Ref	Ref	Ref
Non‐Hispanic Black	−0.241	(−1.614, 1.133)	0.727
Non‐Hispanic White	0.054	(−1.346, 1.453)	0.939
Other Hispanic	−0.539	(−2.040, 0.962)	0.475
Other race—including multi‐racial	−0.872	(−2.295, 0.552)	0.225
Poverty (10,000 dollars)			
> 3	Ref	Ref	Ref
1.1–3	−1.052	(−1.858, −0.247)	0.002
0–1	−1.826	(−2.950, −0.703)	0.011
Educational level			
College or above	Ref	Ref	Ref
High school or equivalent	0.312	(−0.967, 1.591)	0.627
Less than high school	−1.238	(−2.512, 0.036)	0.057
BMI	−0.012	(−0.082, 0.058)	0.725
HbA1c	−0.002	(−0.304, 0.299)	0.987
eGFR	0.013	(−0.009, 0.035)	0.233
ACR	0.000	0.000 (0.000, 0.001)	0.381
Hyperlipidemia			
No	Ref	Ref	Ref
Yes	−0.428	(−1.724, 0.868)	0.511
Hypertension			
No			
Yes	0.058	(−1.158, 1.274)	0.924
Antidiabetic drugs			
None	n	Ref	Ref
OHAS	0.526	(−0.574, 1.627)	0.342
Insulin	1.954	(0.633, 3.276)	0.004
OHAS + Insulin	1.500	(0.054, 2.947)	0.042

Abbreviations: ACR, albumin‐creatinine ratio; BMI, body mass index; CI, confidence interval; e‐GFR, estimated glomerular filtration rate; LCDS, low‐carbohydrate‐diet score; OHAS, oral hypoglycaemic agents; T2D, type 2 diabetes.

### 3.3 The Association Between LCDS and the Prevalence of DKD in T2DM Patients

#### 3.3.1 RCS Fitting the Dose–Response Relationship Between LCDS and the Prevalence of DKD

**FIGURE 2:**
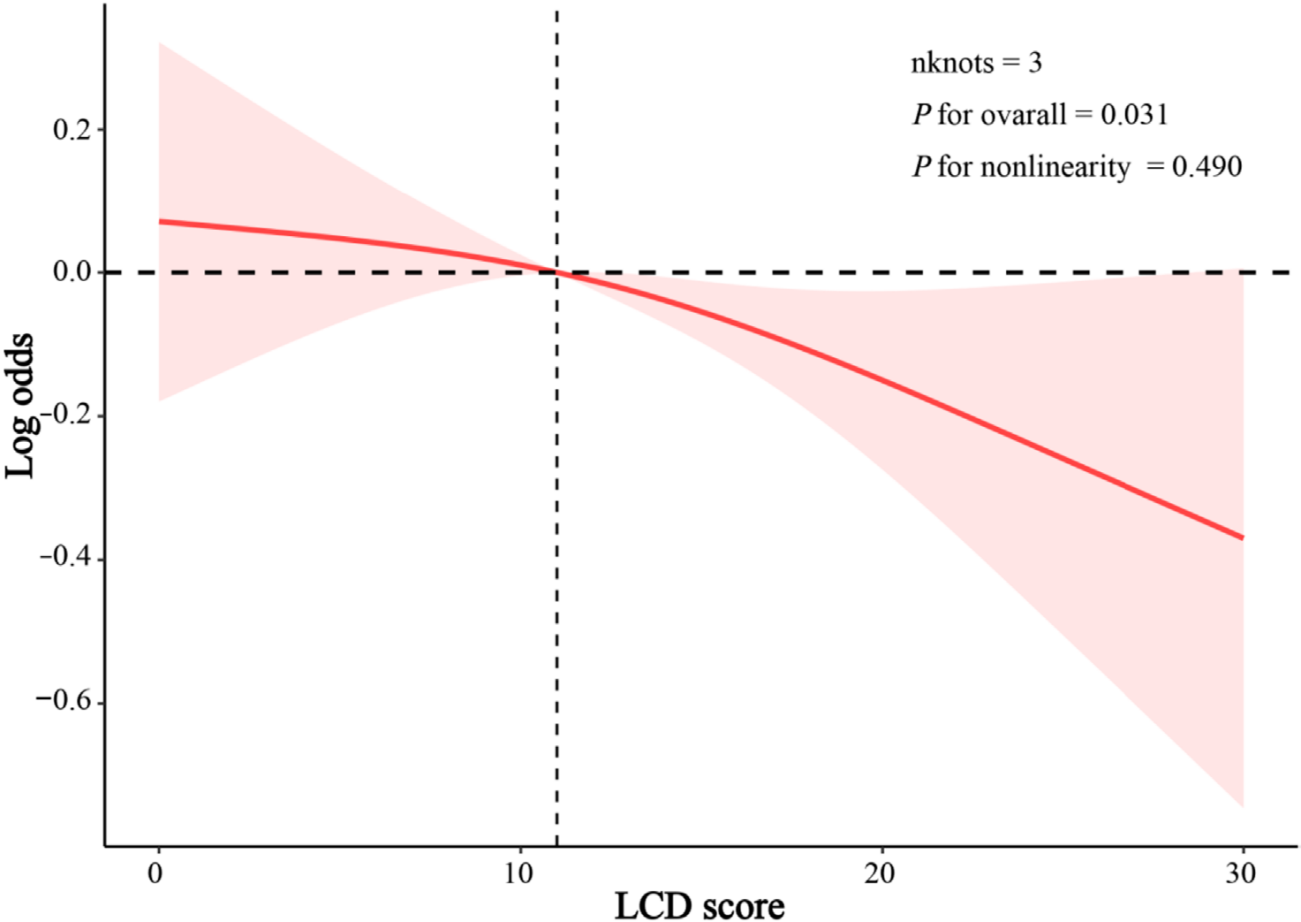
The dose–response relationship between LCDS and DKD in T2D: a restricted cubic spline analysis. Warning against over‐interpreting the high‐score tail.

The dose–response relationship between LCDS and the risk of DKD in T2DM patients was analyzed using RCS. After multivariable adjustment, the findings demonstrated that as LCDS increased, the prevalence of DKD decreased (nknot = 3; *p* for all = 0.031; *p* for nonlinearity = 0.490; Figure 2, Table S5).

#### 3.3.2 Multi‐Factor Logistic Regression Model Assessing the Relationship Between LCDS and the Prevalence of DKD

**FIGURE 3:**
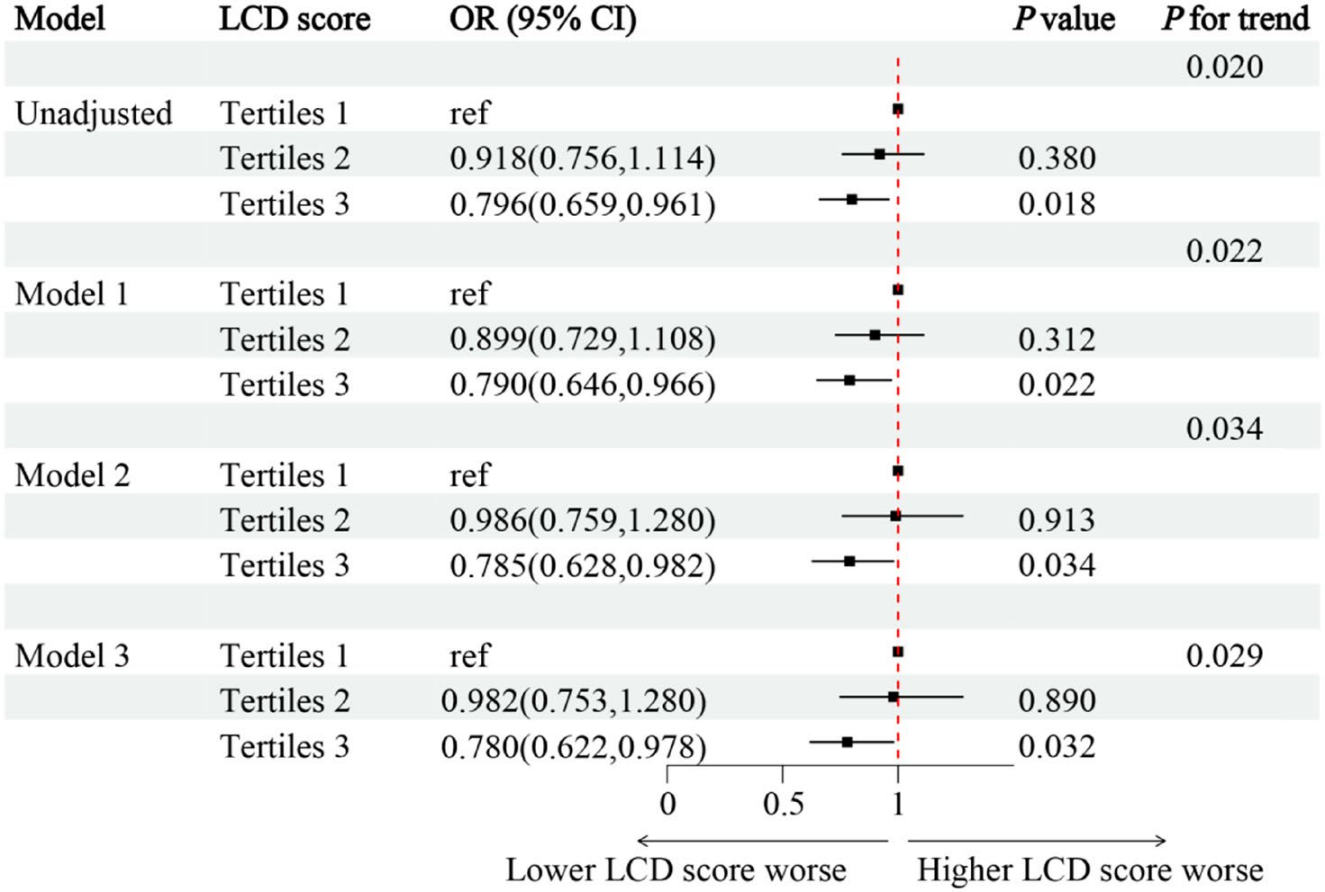
Associations between LCDS and the Prevalence of DKD in individuals with T2D. Model 1 adjusted for baseline age, sex (“Female,” “Male”), race (“Mexican American,” “Non‐Hispanic Black,” “Non‐Hispanic White,” “Other Hispanic,” “Other Race—Including Multi‐Racial”), BMI; Model 2 adjusted for covariates in model 1 plus, smoking (“yes” or “no”), alcohol use (“yes” or “no”), education (“College or above,” “High school or equivalent,” “Less than high school,”) poverty (“0–1,” “1.1–3,” “> 3”), HbA1c, antidiabetic drugs (“None,” “OHAS,” “Insulin,” “OHAS + Insulin”); Model 3^b^ adjusted for covariates in model 2 plus hyperlipidemia (“yes” or “no”), hypertension (“yes” or “no”). ACR, albumin‐creatinine ratio; BMI, body mass index; DKD, diabetes kidney disease; e‐GFR, estimated glomerular filtration rate; LCDS, low‐carbohydrate‐diet score; OHAS, Oral hypoglycaemic agents; T2D, type 2 diabetes.

The relationship between LCDS and the prevalence of DKD in T2DM patients was evaluated using a multi‐factor logistic regression model. Multicollinearity was assessed for all covariates in the multivariable model by examining the VIFs. The maximum VIF was 4.38, associated with the “Race” variable. This value is below the widely accepted threshold of 5 (and the more lenient threshold of 10), suggesting that multicollinearity did not significantly influence the stability or interpretability of our parameter estimates. Complete VIF values were detailed in Table S6. The results indicated that, when unadjusted for variables, patients in Tertiles 3 group experienced a 20.4% lower prevalence of DKD compared to those in Tertiles 1 group (OR = 0.796; 95% CI, 0.659–0.961, *p* = 0.018). After adjusting for baseline age, sex (“Female,” “Male”), race (“Mexican American,” “Non‐Hispanic Black,” “Non‐Hispanic White,” “Other Hispanic,” “Other Race—Including Multi‐Racial”), BMI in model 1, it was observed that patients in Tertiles 3 group had a 21.0% lower prevalence of DKD relative to those in Tertiles 1 group (OR = 0.790; 95% CI, 0.646–0.966, *p* = 0.022). In model 2, which included adjustments for smoke (“yes” or “no”), alcohol use (“yes” or “no”), education (“College or above,” “High school or equivalent,” “Less than high school”), PIR (“0–1,” “1.1–3,” “> 3”), HbA1c, and antidiabetic drugs (“None,” “OHAS,” “Insulin,” “OHAS + Insulin”), patients in Tertiles 3 group again demonstrated a 21.5% lower prevalence of DKD compared to those in Tertiles 1 group (OR = 0.785; 95% CI, 0.628–0.982, *p* = 0.034). Model 3, which further adjusted for hypertension (“yes” or “no”) and hyperlipidemia (“yes” or “no”), revealed that patients in Tertiles 3 group had a 22.0% lower prevalence of DKD when compared to those in Tertiles 1 group (OR = 0.780; 95% CI, 0.622–0.978, *p* = 0.032; Figure 3, Tables S7 and S8).

#### 3.3.3 Stratified Analysis

**FIGURE 4:**
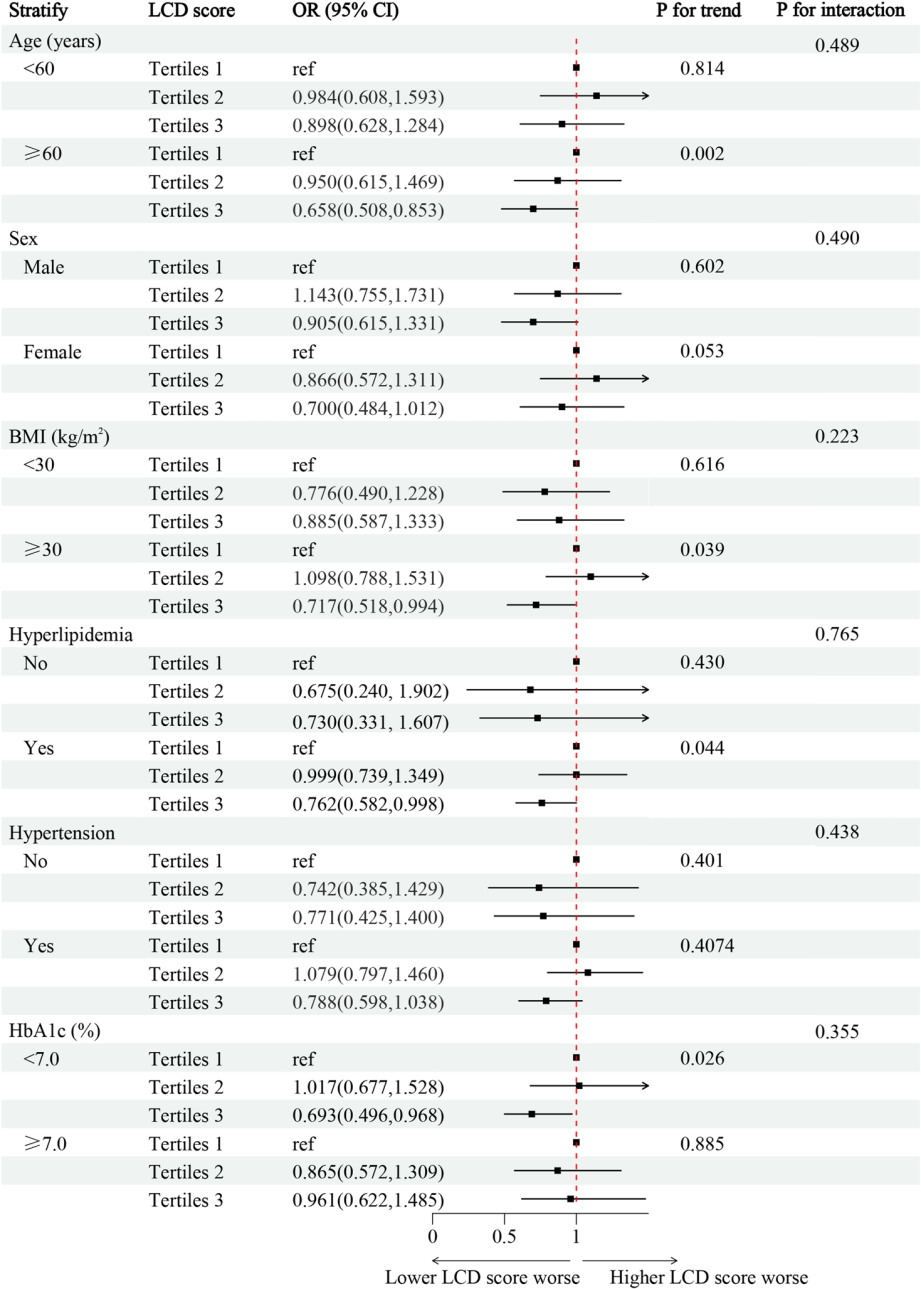
Stratified Analysis of the Association Between LCDS and the Prevalence of DKD in T2D Patients. Adjust for age, sex (“Female,” “Male,”) race (“Mexican American,” “Non‐Hispanic Black,” “Non‐Hispanic White,” “Other Hispanic,” “Other Race—Including Multi‐Racial”), BMI, smoking (“yes” or “no,”) alcohol use (“yes” or “no,”) HbA1c, education (“College or above,” “High school or equivalent,” “Less than high school”), poverty (“0–1,” “1.1–3,” “> 3,”) HbA1c, antidiabetic drugs (“None,” “OHAS,” “Insulin,” “OHAS + Insulin”), hyperlipidemia (“yes” or “no”), hypertension (“yes” or “no”). ACR, albumin‐creatinine ratio; BMI, body mass index; DKD, diabetes kidney disease; e‐GFR, estimated glomerular filtration rate; LCDS, low‐carbohydrate‐diet score; OHAS, oral hypoglycaemic agents; T2D, type 2 diabetes.

A stratified analysis was also conducted based on multivariable adjustment. Among participants aged ≥ 60 years, the T3 group exhibited a 34.2% lower prevalence of DKD compared to T1 (OR = 0.658, 95% CI: 0.508–0.853), whereas no significant trend was observed in those < 60 years (*p* for trend = 0.814), with no interaction with age (*p* for interaction = 0.489). Similarly, in individuals with BMI ≥ 30 kg/m^2^, T3 was associated with a 28.3% lower prevalence (OR = 0.717, 95% CI: 0.518–0.994) versus T1, while no trend emerged in the BMI < 30 kg/m^2^ subgroup (*p* for trend = 0.616) and no BMI‐based interaction existed (*P* for interaction = 0.223). For HbA1c < 7.0%, T3 showed a 30.7% lower prevalence of DKD (OR = 0.693, 95% CI: 0.496–0.968) relative to T1, though no trend was seen in HbA1c ≥ 7.0% (*p* for trend = 0.885) and no interaction was detected (*p* for interaction = 0.355). In hyperlipidemia subgroups, T3 is associated with a lower prevalence of DKD by 23.8% (OR = 0.762, 95% CI: 0.582–0.998) among those with hyperlipidemia, with no trend in non‐hyperlipidemia participants (*p* for trend = 0.430) and no interaction (*p* for interaction = 0.765). Gender and hypertension no significant trends (males: *p*‐trend = 0.602; females: *p* for trend = 0.053; non‐hypertensive: *p* for trend = 0.401; hypertensive: *p* for trend = 0.074) and no interaction (gender: *p* for interaction = 0.490; hypertension: *p* for interaction = 0.438, Figure 4, Table S9).

#### 3.3.4 Sensitivity Analysis

To reduce the potential impact of data imputation on the results, we performed a complete‐case analysis to assess the relationship between LCD and the prevalence of DKD among individuals with T2DM. In the multivariable‐adjusted logistic regression analysis, compared with patients in Tertile 1 group, those in Tertile 3 group exhibited a 24.2% lower prevalence of DKD (OR = 0.758, 95% CI: 0.604 to 0.951; Table S10). This result was consistent with the findings from the multiple imputation‐based multivariable logistic regression analysis.

## 4 Discussion

Our study revealed a linear inverse association between LCDS and the prevalence of DKD among individuals with T2DM, with higher LCDS corresponding to a progressively lower risk of DKD. Compared to those in the lowest LCDS tertile, participants in the highest tertile experienced a 22% reduction in DKD risk. This association remained consistent across subgroups defined by sex, ethnicity, education level, and other potential confounding factors. Furthermore, age, BMI, and blood pressure were identified as potential modifiers of LCDS in this T2DM population.

Accumulating evidence demonstrates a protective effect of a LCD against the progression of DKD. A case–control study utilizing LCDS as the dietary assessment tool, which enrolled 105 female patients with early‐stage DKD, revealed an inverse correlation between LCDS and DKD progress risk (Hajishizari et al. 2023). The DIRECT study addressed the gap in male data through a randomized controlled trial including 318 participants (86% male, 45 with T2DM). This two‐year intervention compared strict carbohydrate‐restricted LCD with Mediterranean diet (MD) and low‐fat diet (LFD) effects on renal function in CKD. Results demonstrated comparable eGFR improvements across LCD, MD, and LFD groups, regardless of CKD stage or T2DM status (Tirosh et al. 2013). Collectively, these two studies suggest sex does not significantly modify the association between carbohydrate‐restricted diets and DKD. Furthermore, a 7‐year retrospective study implementing LCD intervention for approximately 30 months in 143 early‐stage DKD patients showed improvements in creatinine, eGFR, and urine protein‐creatinine ratio. The mean eGFR improvement of 2.4 mL/min/1.73 m^2^ was considered significant when accounting for temporal factors (Unwin et al. 2021). This evidence indicates that low‐carbohydrate, high‐protein, high‐fat dietary patterns can improve clinical outcomes in DKD. These results collectively suggest LCD involvement in DKD pathogenesis and progression. However, our study elucidates the relationship between the LCDS and the prevalence of DKD in patients with T2DM, primarily focusing on the potential association of LCDS with the occurrence of complications in T2DM patients. The results demonstrate an inverse association between LCDS and DKD prevalence, with a higher prevalence of DKD observed in patients with lower LCDS. Compared to the T1 group, patients in the higher LCDS T3 group showed a 22% reduction in DKD prevalence. Additionally, a consistent association was observed in the subgroup analysis based on patient sex. Importantly, consistent results across different HbA1c subgroups suggest this relationship is independent of glycemic control levels, implying potential benefits of LCD for patients across varying glycemic statuses.

The potential mechanisms underlying LCDS‐mediated improvement in DKD prevalence may involve multiple pathways. Firstly, regarding metabolic improvement and weight control: Given that obesity itself constitutes an independent risk factor for CKD (Hojs et al. 2023; Hsu et al. 2006), weight management achieved through LCD represents an essential renoprotective mechanism (Morales et al. 2003). LCD induces “metabolic switching” by promoting gluconeogenesis and ketogenesis (Veech 2004) while inhibiting lipogenesis and enhancing fat mobilization (Tian et al. 2024), complemented by dietary fat's suppression of endogenous cholesterol synthesis (Bosse et al. 2013). These processes collectively contribute to lipid reduction and weight control. Moreover, although LCD often includes high‐fat foods like nuts, studies indicate they do not promote weight gain but may improve blood cholesterol profiles.

Secondly, LCD helps improve blood pressure and glycemic parameters. Our study also identified superior blood pressure and glycemic parameters in patients with higher LCDS. Hemodynamic alterations (glomerular hypertension) and metabolic disturbances (hyperglycemia) are well‐established as two primary determinants in the pathogenesis and progression of DKD (Hostetter 1994; Stratton et al. 2000). Intensive glycemic management has been proven to reduce DKD incidence and delay its progression (Fu et al. 2013). Dietary carbohydrates serve as the primary source of circulating glucose and key stimulants for insulin secretion, exhibiting greater glycemic effects than equivalent amounts of fat or protein (Brouns 2018). By restricting carbohydrate intake, LCD stimulates gluconeogenesis and ketogenesis (Fujita et al. 2020), thereby reducing postprandial glucose fluctuations and insulin requirements while mitigating persistent hyperglycemic damage to glomeruli and renal tubules. Additionally, glomerular hypertension represents another crucial hemodynamic factor in DKD development and progression (Paoli et al. 2013). Beyond significantly lowering HbA1c and fasting blood glucose (Wang et al. 2018) to directly alleviate hyperglycemia‐induced renal injury, LCD may also ameliorate glomerular hypertension through improved insulin sensitivity and resistance promoting urinary sodium excretion (Goldenberg et al. 2021; Paoli et al. 2013), enhanced endothelial function (Unwin et al. 2019), among other mechanisms. However, this mechanism requires further direct evidence, as the observed blood pressure improvements might also result from weight loss, reduced adiposity, and better control of other cardiovascular risk factors.

Finally, LCD exerts benefits through antioxidant stress and mitochondrial protection pathways. As a highly metabolically active organ, the kidney exhibits exceptionally high oxygen consumption and possesses a rich mitochondrial network second only to the myocardium in density (Wang et al. 2010). Mitochondrial dysfunction has been identified as a key pathological alteration occurring early in DKD (Forbes and Thorburn 2018). Under hyperglycemic conditions, the oxidant‐antioxidant balance is disrupted, mitochondrial oxidative phosphorylation is impaired, and reactive oxygen species generation increases. LCD has been shown to upregulate various antioxidant proteins such as NADPH, NQO1, and SOD (Greco et al. 2016; Roberts et al. 2017) and improve impaired mitochondrial function and biogenesis in diabetes by activating PGC‐1α and SIRT1 signaling pathways (Roberts et al. 2017), thereby counteracting mitochondrial dysfunction present early in DKD (Forbes and Thorburn 2018) and establishing an additional protective mechanism.

While our study demonstrates an association between a LCD and a lower prevalence of DKD, it is critical to consider the dietary composition of an LCD. The potential long‐term health implications can vary substantially depending on the primary food sources of fat and protein. An LCD pattern rich in animal fats and processed meats may lead to increased intake of saturated fats and cholesterol, which could elevate cardiovascular risk. Concurrently, a reduction in carbohydrate intake, if not carefully planned, can result in insufficient dietary fiber, potentially adversely affecting gut microbiota and overall metabolic health (Murtaza et al. 2019; Muscogiuri et al. 2019; Barber et al. 2021). Given the demonstrated superiority of plant‐based diets in delaying the progression of CKD (Naber and Purohit 2021; Dang et al. 2025). Therefore, we wish to emphasize the concept of a high‐qualityplant‐based LCD. This approach prioritizes the reduction of refined carbohydrates and sugars while encouraging the consumption of nutrient‐dense, plant‐derived foods. Such a diet includes healthy fats from sources like nuts, seeds, avocados, and olive oil, and proteins from legumes and soy products. These choices are associated with higher fiber intake, unsaturated fats, and various phytonutrients, which may confer additional benefits for glycemic control, lipid profiles, and inflammation, thereby potentially mitigating the risks associated with a poorly constructed LCD. Future dietary guidelines and clinical recommendations should stress the importance of promoting this balanced, plant‐predominant LCD pattern over versions high in animal products to maximize benefits and minimize risks for patients with diabetes at risk for DKD. However, the specific value and mechanisms of a plant‐based low‐carbohydrate diet require more evidence‐based research to clarify.

Although a statistically significant association was observed between LCDS and DKD prevalence (OR = 0.78), the modest absolute risk difference of approximately 2%–3% across LCD score tertiles suggests that, at the individual patient level, the immediate clinical impact of adopting a LCD as a standalone intervention might be limited. while the intervention is more efficient when targeted specifically to the high‐risk subgroup (NNT = 33–50), it remains potentially valuable at the population level (NNT = 105–158), particularly for a significant clinical outcome. The population‐level NNT provides a realistic estimate of the effort required for broader implementation and helps contextualize the public health impact of our findings. Clinicians should consider this effect alongside other critical factors such as the patient's overall dietary pattern, preferences, the potential costs and burdens of dietary intervention, and the management of other cardiovascular risk factors. However, from a public health perspective, even a marginal reduction in DKD prevalence could translate into a substantial number of preventable cases when applied across the large population of individuals with T2DM. Consequently, integrating individualized LCD guidance into multifaceted diabetes management strategies holds significant potential population health value. This potential population‐level benefit may justify further investigation into how dietary guidance could be integrated into broader, multi‐faceted diabetes management strategies.

This study has several limitations that should be considered when interpreting the findings. First, the possibility of residual confounding cannot be excluded, despite our extensive adjustments for known covariates. Second, the LCDS, though derived using rigorous criteria, relies on dietary recall data that are inherently subject to measurement error and a degree of subjectivity. Most importantly, the cross‐sectional design fundamentally limits causal inference. The observed association between LCDS and DKD could be influenced by reverse causality, wherein the presence of DKD itself may lead to dietary modifications. Therefore, our results, while indicating a statistically significant association, do not establish causality. Finally, the NHANES database primarily collects data from community‐based general populations and does not systematically gather variables necessary to identify conditions such as urinary tract infections, macrohematuria, or “severe illness within the past 3 months.” Consequently, we were unable to exclude participants with these conditions to assess the potential confounding effects of acute kidney injury (AKI) on the study findings. Future investigations employing long‐term prospective cohort studies or randomized controlled trials are warranted to verify our findings and elucidate the causal direction of this relationship.

In summary, this study demonstrates an inverse association between a higher LCDS and the prevalence of DKD in individuals with T2DM. The findings suggest that a dietary pattern with moderate carbohydrate restriction, implemented under medical supervision, may contribute to improved renal outcomes in diabetic patients, offering a potential new direction for dietary recommendation. Notwithstanding these observations, the limitations of the present work, particularly its cross‐sectional nature, highlight the necessity for future prospective research to verify this association and explore the long‐term impact of LCDs on DKD risk in T2DM.

## Author Contributions

Conception and design of the study: Wuyu Gao, Rong Ma, Hui Gu, Yun Sun, Xiang Xiao. Acquisition and analysis of data: Wuyu Gao, Rong Ma, Yun Sun, Xiang Xiao, Hui Gu. Drafting the manuscript or figures: Wuyu Gao, Rong Ma, Hui Gu, Hao Liu, Mao Xiao, Xue Zhang, Zhuoxing Li, Yun Sun, Xiang Xiao. Funding: Xiang Xiao. Statistical analysis and language polishing: Yiting Wang.

## Funding

This work was supported by The Science and technology fund of Chengdu Medical College (CYZYB22‐02); The research fund of Sichuan Medical and Health Care Promotion institute (KY2022QN0309); Sichuan Provincial Medical Association Youth Innovation Project (Q23021); Health Commission of Sichuan Province Medical Science and Technology Program (24QNMP100); The National Natural Science Foundation of China (82200815). The funder had no role in the study design, data collection and analysis, decision to publish, or preparation of the manuscript.

## Ethics Statement

The study protocol conformed to the ethical standards of the 1964 Declaration of Helsinki and its subsequent amendments, with approval from the National Committee for Ethical Review of Health Statistics Research and signed informed consent from all participants.

## Conflicts of Interest

The authors declare no conflicts of interest.

## Supporting information


**Data S1:** supporting Information.

## Data Availability

Some or all datasets generated during and/or analyzed during the current study are not publicly available but are available from the corresponding author on reasonable request.
