# Supplementary material for: The Relationship Between a Low‐Carbohydrate Diet and the Prevalence of Diabetic Kidney Disease in Patients With Type 2 Diabetes: A Cross‐Sectional Study
Source: Food Sci Nutr. 2026 Mar 19;14(3):e71462. doi: 10.1002/fsn3.71462 (PMC13093690; doi:10.1002/fsn3.71462)
Supplement: Supplementary file 1 — Data S1: supporting Information. [file FSN3-14-e71462-s001.docx]

**Supplementary materials**

**Supplementary -table 1.** Definitions/criteria of Some Diagnoses

| Variables | Definitions/criteria |
| --- | --- |
| Smoker | Smoking more than 100 cigarettes in previous and now. |
| Alcohol user ^[1]^ | ≥2 drinks per day for females, ≥3 drinks per day for males, or binge drinking ≥2 days per month.  Binge drinking (≥4 drinks on the same occasion for females, ≥5 drinks on the same occasion for males) on 5 or more days per month. |
| Hypertension ^[2]^ | 1. Self-reported hypertension diagnosis, (2) Use of anti-hypertensive medication, (3) Average systolic blood pressure (SBP) > 140 mmHg, (4) Average diastolic blood pressure (DBP) > 90 mmHg, meet any of the above conditions. |
| Hyperlipidemia | (1) Triglyceridemia ≥ 150 mg/dl; (2) Hypercholesterolemia: a) total cholesterol ≥ 200 mg/dl, b) low-density lipoprotein ≥ 130 mg/dl), c). high-density lipoprotein (< 40 mg/dl, male; < 50 mg/dl, female), meet any of the above conditions; (3) Use of lipid-lowering drugs; meet any of the above conditions. |

**References**

[1] RATTAN P, PENRICE D D, AHN J C, et al. Inverse Association of Telomere Length With Liver Disease and Mortality in the US Population [J]. Hepatology communications, 2022, 6(2): 399-410.

[2] WHELTON P K, CAREY R M, ARONOW W S, et al. 2017 ACC/AHA/AAPA/ABC/ACPM/AGS/APhA/ASH/ASPC/NMA/PCNA Guideline for the Prevention, Detection, Evaluation, and Management of High Blood Pressure in Adults: A Report of the American College of Cardiology/American Heart Association Task Force on Clinical Practice Guidelines [J]. Journal of the American College of Cardiology, 2018, 71(19): e127-e248.

**Supplementary -table 2.** Calculation of LCDS

| Points | Carbohydrate/ total energy, % | Protein/ total energy, % | Fat/ total energy, % |
| --- | --- | --- | --- |
| 0 | > 56.0 | <14.1 | <26.0 |
| 1 | 51.6-56.0 | 14.1-15.6 | 26.0-29.5 |
| 2 | 49.1-51.5 | 15.7-16.6 | 29.6-31.6 |
| 3 | 47.1-49.0 | 16.7-17.3 | 31.7-33.2 |
| 4 | 45.2-47.0 | 17.4-18.0 | 33.3-34.7 |
| 5 | 43.3-45.1 | 18.1-18.7 | 34.8-36.1 |
| 6 | 41.2-43.2 | 18.8-19.4 | 36.2-37.7 |
| 7 | 38.8-41.1 | 19.5-20.3 | 37.8-39.5 |
| 8 | 35.4-38.7 | 20.4-21.5 | 39.6-42.0 |
| 9 | 29.3-35 .3 | 21.6-24.0 | 42.1-46.9 |
| 10 | <29.3 | >24.0 | >46.9 |

LCDS is the sum of the scores for the three nutrients, ranging from 0 to 30. LCDS, low-carbohydrate-diet score.

**Supplementary -table 3.** Baseline Clinical Features of Enrolled Individuals with T2D Before and After Multiple Imputation

| Variable | Before multiple imputation | After multiple imputation | SMD (%) |
| --- | --- | --- | --- |
| LCDS | 12.058(0.152) | 12.058(0.152) | 0.00 |
| Age (years) | 59.872(0.328) | 59.872(0.328) | 0.00 |
| Sex (%) |  |  | 0.00 |
| Female | 48.801(0.019) | 48.801(0.019) |  |
| Male | 51.199(0.018) | 51.199(0.018) |  |
| Race (%) |  |  | 0.00 |
| Mexican American | 10.350(0.013) | 10.350(0.013) |  |
| Non-Hispanic Black | 13.756(0.010) | 13.756(0.010) |  |
| Non-Hispanic White | 60.070(0.031) | 60.070(0.031) |  |
| Other Hispanic | 6.413(0.006) | 6.413(0.006) |  |
| Other Race - Including Multi-Racial | 9.411(0.007) | 9.411(0.007) |  |
| PIR (10,000 dollars) (%) |  |  | 9.68 |
| >3 | 38.874(0.017) | 20.503(0.011) |  |
| 1.1-3 | 38.236(0.017) | 38.497(0.017) |  |
| 0-1 | 15.630(0.009) | 41.001(0.018) |  |
| Educational level (%) |  |  | 0.10 |
| College or above | 9.545(0.008) | 9.545(0.008) |  |
| High school or equivalent | 37.932(0.018) | 37.936(0.018) |  |
| Less than high school | 52.445(0.019) | 52.519(0.019) |  |
| BMI (%) |  |  | 0.60 |
| Underweight | 0.265(0.001) | 0.265(0.001) |  |
| Normal weight | 10.785(0.007) | 10.901(0.007) |  |
| Overweight | 24.995(0.010) | 25.450(0.010) |  |
| Obesity | 62.742(0.023) | 63.384(0.023) |  |
| Alcohol use (%) |  |  | 3.39 |
| No | 13.335(0.008) | 15.400(0.008) |  |
| Yes | 77.931(0.026) | 84.600(0.026) |  |
| Smoke (%) |  |  | < 0.01 |
| No | 50.207(0.014) | 50.233(0.015) |  |
| Yes | 49.767(0.021) | 49.767(0.021) |  |
| e-GFR (ml/min/1.73m^2^) | 83.491(0.538) | 83.369(0.537) | 3.39 |
| e-GFR (ml/min/1.73m2) (%) |  |  | 0.30 |
| ≥60 | 81.242(0.026) | 81.291(0.026) |  |
| <60 | 17.760(0.010) | 18.709(0.010) |  |
| ACR (mg/g) | 108.671(8.897) | 134.608(11.767) | 2.04 |
| ACR (mg/g) (%) |  |  | 1.60 |
| <30 | 73.312(0.023) | 73.521(0.023) |  |
| 30-60 | 19.706(0.011) | 19.807(0.010) |  |
| ≥300 | 6.153(0.006) | 6.672(0.006) |  |
| HbA1c (%) | 7.073(0.033) | 7.066(0.033) | 0.00 |
| Hyperlipidemia (%) |  |  | <0.01 |
| No | 11.472(0.007) | 11.472(0.007) |  |
| Yes | 88.520(0.027) | 88.528(0.027) |  |
| Hypertension (%) |  |  | 0.00 |
| No | 29.006(0.015) | 29.006(0.015) |  |
| Yes | 70.994(0.022) | 70.994(0.022) |  |
| Antidiabetic drugs (%) |  |  | 8.95 |
| None | 21.405(0.012) | 23.822(0.012) |  |
| OHAS | 43.463(0.015) | 51.362(0.017) |  |
| Insulin | 6.239(0.005) | 9.134(0.007) |  |
| OHAS + Insulin | 10.950(0.007) | 15.682(0.010) |  |
| Energy intake (kca/day) | 1951.406(22.477) | 1951.406(22.477) | 0.00 |

LCDS, low-carbohydrate-diet score; PIR, poverty-income ratio; BMI, Body Mass Index; ACR, albumin-creatinine ratio; e-GFR, estimated glomerular filtration rate; OHAS, Oral hypoglycaemic agents; SMD, Standardized Mean Difference.

**Supplementary -table 4.** Current Status of LCDS in T2D Patients Over Time

| Variables | Total | Years | | | | | *P*-value |
| --- | --- | --- | --- | --- | --- | --- | --- |
|  |  | 2009-2010 | 2011-2012 | 2013-2014 | 2015-2016 | 2017-2018 |  |
|  |  |  |  |  |  |  | 0.076 |
| T1  (0≤LCDS≤7) | 16.863(0.008) | 17.708(0.015) | 19.580(0.011) | 23.697(0.017) | 22.153(0.011) | 16.863(0.008) |  |
| T2  (7＜LCDS≤15) | 17.492(1.437) | 17.928(1.691) | 20.549(1.238) | 21.270(1.909) | 22.760(1.464) | 17.492(1.437) |  |
| T3  (15≤LCDS<30) | 18.517(1.536) | 17.975(1.686) | 19.060(1.339) | 25.923(1.599) | 18.526(1.672) | 18.517(1.536) |  |

T2D, type 2 diabetes; LCDS, low-carbohydrate-diet score.

**Supplementary -table 5.** Distribution of LCDS Score Categories with Sample Sizes and Weighted Percentages

| LCS Interval | N | Weighted (%) |
| --- | --- | --- |
| <10 | 1963 | 39.5 |
| 10-15 | 976 | 22,5 |
| 15-20 | 806 | 19.4 |
| 20-25 | 576 | 13.3 |
| >25 | 237 | 5.3 |
| Total | 4558 | 100 |

LCDS, low-carbohydrate-diet score.

**Supplementary-table 6.** Variance Inflation Factors (VIFs) for Predictor Variables in the Multivariable Model

| Variable | VIF |
| --- | --- |
| Age | 1.95 |
| **HbA1c** | 1.93 |
| **Sex** | 2.19 |
| **Race** | 4.38 |
| **BMI** | 3.31 |
| **Alcohol user** | 1.67 |
| **Smoke** | 1.43 |
| **Educational** | 2.99 |
| **PIR** | 3.62 |
| **Hypertension** | 1.93 |
| **Hyperlipidemia** | 1.81 |
| **Antidiabetic** | 4.11 |

PIR, poverty-income ratio; BMI, Body Mass Index; ACR, albumin-creatinine ratio; e-GFR, estimated glomerular filtration rate.

**Supplementary -table 7.** Logistic-regression analysis of the Association of LCDS with DKD Prevalence In Patient with T2D

| Variables | Unadjusted | | Model 3 | |
| --- | --- | --- | --- | --- |
|  | OR (95% CI) | *P*-value | OR (95% CI) | *P*-value |
| LCDS |  |  |  |  |
| T1 | ref |  | ref |  |
| T2 | 0.918(0.756,1.114) | 0.380 | 0.982(0.753,1.280) | 0.89 |
| T3 | 0.796(0.659,0.961) | **0.018** | 0.780(0.622,0.978) | **0.032** |
| Age | 1.053(1.046,1.062) | <0.001 | 1.057(1.047,1.068) | **<0.001** |
| Sex |  |  |  |  |
| Female | ref |  | ref |  |
| Male | 0.993(0.796,1.240) | 0.952 | 1.105(0.838,1.457) | 0.470 |
| Race |  |  |  |  |
| Mexican American | ref |  | ref |  |
| Non-Hispanic Black | 1.265(1.000,1.600) | **0.050** | 0.993(0.688,1.433) | 0.969 |
| Non-Hispanic White | 1.210(0.975,1.502) | 0.082 | 0.982(0.678,1.420) | 0.92 |
| Other Hispanic | 0.831(0.620,1.112) | 0.209 | 0.672(0.417,1.083) | 0.101 |
| Other Race - Including Multi-Racial | 1.054(0.767,1.449) | 0.744 | 0.891(0.539,1.473) | 0.647 |
| BMI |  |  |  |  |
| Underweight | ref |  | ref |  |
| Normal weight | 0.697(0.229,2.122) | 0.521 | 0.277(0.092,0.835) | **0.023** |
| Overweight | 0.530(0.172,1.629) | 0.263 | 0.178(0.059,0.533) | **0.003** |
| Obesity | 0.607(0.200,1.840) | 0.372 | 0.296(0.101,0.868) | **0.027** |
| Alcohol use |  |  |  |  |
| No | ref |  | ref |  |
| Yes | 0.692(0.552,0.868) | **0.002** | 0.852(0.610,1.190) | 0.340 |
| Smoke |  |  |  |  |
| No | ref |  | ref |  |
| Yes | 1.243(1.024,1.509) | 0.028 | 1.115(0.841,1.478) | 0.442 |
| Hyperlipidemia |  |  |  |  |
| No | ref |  | ref |  |
| Yes | 1.101(0.835,1.453) | 0.489 | 0.795(0.561,1.127) | 0.193 |
| Hypertension |  |  |  |  |
| No | ref |  | ref |  |
| Yes | 2.348(1.925,2.863) | <0.001 | 1.561(1.190,2.048) | 0.002 |
| Antidiabetic drugs (%) |  |  |  |  |
| None | ref |  | ref |  |
| OHAS | 1.268(1.018,1.578) | **0.034** | 0.860(0.647,1.144) | 0.295 |
| Insulin | 3.337(2.321,4.799) | **<0.001** | 1.597(1.037,2.461) | **0.034** |
| OHAS + Insulin | 2.306(1.649,3.224) | **<0.001** | 1.152(0.793,1.675) | 0.451 |
| HbA1c | 1.216(1.159,1.276) | **<0.001** | 1.254(1.148,1.369) | **<0.001** |
| Poverty (10,000 dollars) |  |  |  |  |
| >3 | ref |  | ref |  |
| 1.1-3 | 1.567(1.206,2.034) | **0.001** | 1.529(1.179,1.984) | **0.002** |
| 0-1 | 1.546(1.253,1.908) | **<0.001** | 1.961(1.371,2.807) | **<0.001** |
| Educational level |  |  |  |  |
| College or above | ref |  | ref |  |
| High school or equivalent | 1.215(0.958,1.541) | 0.107 | 0.961(0.718,1.286) | 0.783 |
| Less than high school | 1.463(1.099,1.948) | **0.010** | 0.864(0.579,1.289) | 0.466 |

DKD, diabetic kidney disease; T2D, type 2 diabetes; OR, Odd Ratio; CI, Confidence Internal; LCDS, low-carbohydrate-diet score; BMI, Body Mass Index; OHAS, Oral hypoglycaemic agents.

**Supplementary -table 8.** The Association of LCDS with DKD Prevalence In Patient with T2D

| Variables | Unadjusted | | Model 1 | | Model 2 | | Model 3 | |
| --- | --- | --- | --- | --- | --- | --- | --- | --- |
|  | OR (95% CI) | *P*-value | OR (95% CI) | *P*-value | OR (95% CI) | *P*-value | OR (95% CI) | *P*-value |
| T1 | ref |  | ref |  | ref |  | ref |  |
| T2 | 0.918(0.756,1.114) | 0.38 | 0.899(0.729,1.108) | 0.312 | 0.986(0.759,1.280) | 0.913 | 0.982(0.753,1.280) | 0.89 |
| T3 | 0.796(0.659,0.961) | 0.018 | 0.790(0.646,0.966) | 0.022 | 0.785(0.628,0.982) | 0.034 | 0.780(0.622,0.978) | 0.032 |
| *P* for trend |  | 0.018 |  | 0.022 |  | 0.031 |  | 0.029 |

**Model 1** adjusted for baseline age, sex, race, BMI; **Model 2** adjusted for covariates in model 1 plus , smoke (‘yes’ or ‘no’), alcohol use (‘yes’ or ‘no’), education (‘College or above’, ‘High school or equivalent’, ‘Less than high school’), poverty (‘0-1’, ‘1.1-3’, ‘＞3’), HbA1c, Antidiabetic drugs (‘None’, ‘OHAS’, ‘Insulin’, ‘OHAS + Insulin’); **Model 3^b^** adjusted for covariates in model 2 plus hyperlipidemia (‘yes’ or ‘no’), hypertension (‘yes’ or ‘no’). LCDS, low-carbohydrate-diet score; T2D, type 2 diabetes; DKD, diabetes kidney disease; OR, Odd Ratio; CI, confidence interval; BMI, Body Mass Index; ACR, albumin-creatinine ratio; e-GFR, estimated glomerular filtration rate; OHAS, Oral hypoglycaemic agents.

**Supplementary -table 9.** Stratified analysis of the Association of LCDS with DKD Prevalence In Patient with T2D

| Variable | OR（95% CI） | | | P for trend | P for interaction |
| --- | --- | --- | --- | --- | --- |
|  | Tertiles 1 | Tertiles 2 | Tertiles 3 |  |  |
| Age (years) |  |  |  |  | 0.489 |
| <60 | ref | 0.984(0.608,1.593) | 0.950(0.615,1.469) | 0.814 |  |
| ≥60 | ref | 0.898(0.628,1.284) | 0.658(0.508,0.853) | **0.002** |  |
| Gender |  |  |  |  | 0.490 |
| Male | ref | 1.143(0.755,1.731) | 0.905(0.615,1.331) | 0.602 |  |
| Female | ref | 0.866(0.572,1.311) | 0.700(0.484,1.012) | 0.053 |  |
| BMI (kg/m^2^) |  |  |  |  | 0.223 |
| <30 | ref | 0.776(0.490,1.228) | 0.885(0.587,1.333) | 0.616 |  |
| ≥30 | ref | 1.098(0.788,1.531) | 0.717(0.518,0.994) | **0.039** |  |
| **HbA1c** |  |  |  |  | 0.355 |
| <7.0 | ref | 1.017(0.677,1.528) | 0.693(0.496,0.968) | **0.026** |  |
| ≥7.0 | ref | 0.865(0.572,1.309) | 0.961(0.622,1.485) | 0.885 |  |
| Hypertension |  |  |  |  | 0.438 |
| No | ref | 0.742(0.385,1.429) | 0.771(0.425,1.400) | 0.401 |  |
| Yes | ref | 1.079(0.797,1.460) | 0.788(0.598,1.038) | 0.074 |  |
| **Hyperlipidemia** |  |  |  |  | 0.765 |
| No | ref | 0.675(0.240, 1.902) | 0.730(0.331, 1.607) | 0.430 |  |
| Yes | ref | 0.999(0.739,1.349) | 0.762(0.582,0.998) | **0.044** |  |

Adjust for age, sex, race, BMI, smoke (‘yes’ or ‘no’), alcohol use (‘yes’ or ‘no’), education (‘College or above’, ‘High school or equivalent’, ‘Less than high school’), poverty (‘0-1’, ‘1.1-3’, ‘＞3’), HbA1c, Antidiabetic drugs (‘None’, ‘OHAS’, ‘Insulin’, ‘OHAS + Insulin’), hyperlipidemia (‘yes’ or ‘no’), hypertension (‘yes’ or ‘no’). LCDS, low-carbohydrate-diet score; T2D, type 2 diabetes; DKD, diabetes kidney disease; OR, Odd Ratio; CI, confidence interval; BMI, Body Mass Index; ACR, albumin-creatinine ratio; e-GFR, estimated glomerular filtration rate; OHAS, Oral hypoglycaemic agents.

**Supplementary -table 10.** Comparison of Multiple Imputation and Complete-Case Analyses for the Association of LCDS with DKD Prevalence in Type 2 Diabetes

| Variables | Multiple Imputation Analysis | | Complete-case analysis | |
| --- | --- | --- | --- | --- |
|  | OR (95% CI) | *P*-value | OR (95% CI) | *P*-value |
| LCDS |  |  |  |  |
| T1 | ref |  | ref |  |
| T2 | 0.982(0.753,1.280) | 0.89 | 0.950(0.734,1.230) | 0.691 |
| T3 | 0.780(0.622,0.978) | 0.032 | 0.758(0.604,0.951) | 0.018 |
| Age | 1.057(1.047,1.068) | <0.001 | 1.059(1.048,1.070) | <0.001 |
| Sex |  |  |  |  |
| Female | ref |  | ref |  |
| Male | 1.105(0.838,1.457) | 0.470 | 1.081(0.826,1.414) | 0.566 |
| Race |  |  |  |  |
| Mexican American | ref |  | ref |  |
| Non-Hispanic Black | 0.993(0.688,1.433) | 0.969 | 1.054(0.763,1.457) | 0.744 |
| Non-Hispanic White | 0.982(0.678,1.420) | 0.920 | 1.010(0.729,1.399) | 0.953 |
| Other Hispanic | 0.672(0.417,1.083) | 0.101 | 0.631(0.417,0.955) | 0.03 |
| Other Race - Including Multi-Racial | 0.891(0.539,1.473) | 0.647 | 0.898(0.572,1.411) | 0.636 |
| BMI |  |  |  |  |
| Underweight | ref |  | ref |  |
| Normal weight | 0.277(0.092,0.835) | 0.023 | 0.268(0.092,0.782) | 0.017 |
| Overweight | 0.178(0.059,0.533) | 0.003 | 0.188(0.064,0.551) | 0.003 |
| Obesity | 0.296(0.101,0.868) | 0.027 | 0.294(0.102,0.847) | 0.024 |
| Alcohol use |  |  |  |  |
| No | ref |  | ref |  |
| Yes | 0.852(0.610,1.190) | 0.340 | 0.873(0.642,1.187) | 0.38 |
| Smoke |  |  |  |  |
| No | ref |  | ref |  |
| Yes | 1.115(0.841,1.478) | 0.442 | 1.180(0.918,1.515) | 0.191 |
| Hyperlipidemia |  |  |  |  |
| No | ref |  | ref |  |
| Yes | 0.795(0.561,1.127) | 0.193 | 0.781(0.563,1.085) | 0.138 |
| Hypertension |  |  |  |  |
| No | ref |  | ref | ref |
| Yes | 1.561(1.190,2.048) | 0.002 | 1.534(1.169,2.014) | 0.003 |
| Antidiabetic drugs (%) |  |  |  |  |
| None | ref |  | ref |  |
| OHAS | 0.860(0.647,1.144) | 0.295 | 0.934(0.716,1.219) | 0.61 |
| Insulin | 1.597(1.037,2.461) | 0.034 | 1.894(1.224,2.932) | 0.005 |
| OHAS + Insulin | 1.152(0.793,1.675) | 0.451 | 1.297(0.913,1.843) | 0.143 |
| HbA1c | 1.254(1.148,1.369) | <0.001 | 1.232(1.137,1.335) | <0.001 |
| Poverty (10,000 dollars) |  |  |  |  |
| >3 | ref |  | ref |  |
| 1.1-3 | 1.529(1.179,1.984) | 0.002 | 1.976(1.426,2.738) | <0.001 |
| 0-1 | 1.961(1.371,2.807) | <0.001 | 1.668(1.323,2.104) | <0.001 |
| Educational level |  |  |  |  |
| College or above | ref |  | ref |  |
| High school or equivalent | 0.961(0.718,1.286) | 0.783 | 0.906(0.692,1.184) | 0.462 |
| Less than high school | 0.864(0.579,1.289) | 0.466 | 0.849(0.589,1.225) | 0.375 |

All analyses adjusted for baseline age, sex, race, BMI, smoke (‘yes’ or ‘no’), alcohol use (‘yes’ or ‘no’), education (‘College or above’, ‘High school or equivalent’, ‘Less than high school’), poverty (‘0-1’, ‘1.1-3’, ‘＞3’), HbA1c, Antidiabetic drugs (‘None’, ‘OHAS’, ‘Insulin’, ‘OHAS + Insulin’), hyperlipidemia (‘yes’ or ‘no’), hypertension (‘yes’ or ‘no’). LCDS, low-carbohydrate-diet score; T2D, type 2 diabetes; DKD, diabetes kidney disease; OR, Odd Ratio; CI, confidence interval; BMI, Body Mass Index; ACR, albumin-creatinine ratio; e-GFR, estimated glomerular filtration rate; OHAS, Oral hypoglycaemic agents.


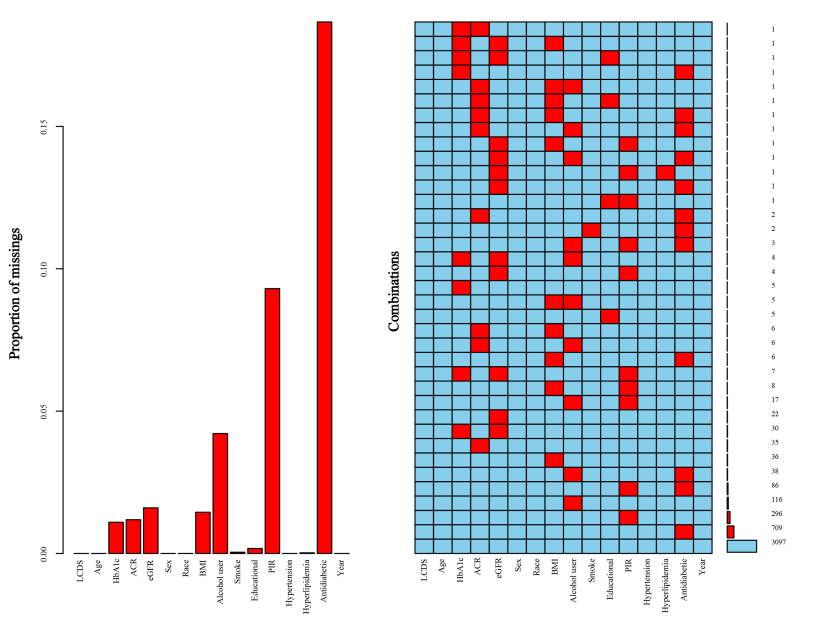


**s-Figure 1.** Missing Data Patterns and Proportions.


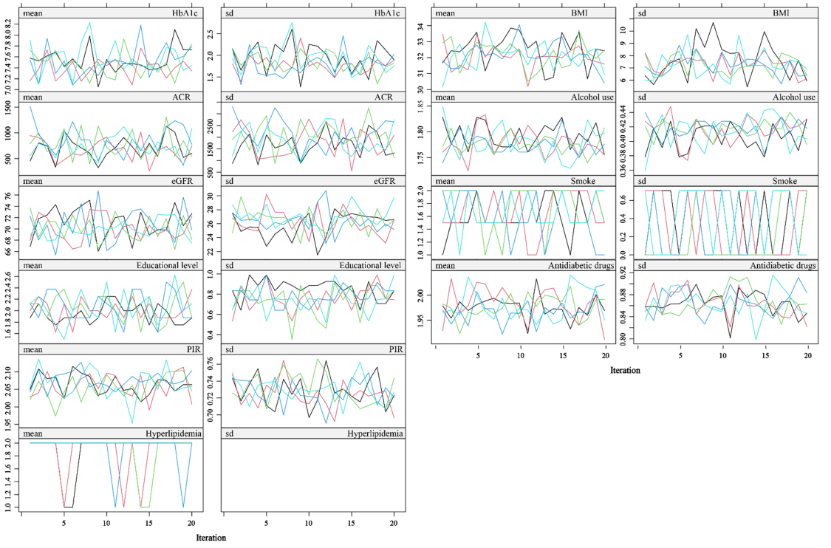


**s-Figure 2.** Convergence Diagnostics for Multiple Imputation: Trace Plots of Means and Standard Deviation.

**R code**

library(nhanesR)

#________________________________________________________1.数据提取

#1.1人口学数据 49693

c1 <- db_demo(ageyr = 'age',sex = "sex",eth1 = 'ethic',eth3 = "ethic3", years = 2009:2018,wtmec2yr = 'wtmec2yr',Year = T)

c1 <- add_col(data = c1,colname = 'age2',value = '0', condition = c1$age < 60)

c1 <- add_col(data = c1,colname = 'age2',value = '1', condition = c1$age >= 60)

c1$age2 <- factor(c1$age2, levels=c('0','1'))

c1 <- add_col(data = c1,colname = 'age3',value = '0', condition = c1$age < 65)

c1 <- add_col(data = c1,colname = 'age3',value = '1', condition = c1$age >= 65)

c1$age3 <- factor(c1$age3, levels=c('0','1'))

unique(c1$ethic3)

#1.2 BMI 47652

c2 <- db_bodyMeasure(height_cm = 'height',Weight_kg = 'weight',BMI_kg.m2 = 'BMI',years = 2009:2018,waist_circumference_cm = T)

c2 <- add_col(data = c2,colname = 'BMI2',value = '0', condition = c2$BMI < 18.5)

c2 <- add_col(data = c2,colname = 'BMI2',value = '1', condition = c2$BMI >= 18.5 & c2$BMI < 25)

c2 <- add_col(data = c2,colname = 'BMI2',value = '2', condition = c2$BMI >= 25 & c2$BMI < 30)

c2 <- add_col(data = c2,colname = 'BMI2',value = '3', condition = c2$BMI >= 30)

c2$BMI2 <- factor(c2$BMI2, levels=c('0','1','2','3'))

c2 <- add_col(data = c2,colname = 'BMI3',value = '0', condition = c2$BMI < 30)

c2 <- add_col(data = c2,colname = 'BMI3',value = '1', condition = c2$BMI >= 30)

c2$BMI3 <- factor(c2$BMI3, levels=c('0','1'))

c2 <- add_col(data = c2,colname = 'BMI4',value = '0', condition = c2$BMI < 25)

c2 <- add_col(data = c2,colname = 'BMI4',value = '1', condition = c2$BMI >= 25)

c2$BMI4 <- factor(c2$BMI4, levels=c('0','1'))

#1.3 饮酒 28866

c3 <- diag_alcohol.user(years = 2009:2018)

c3 <- add_col(data = c3,colname = 'alcohol.user2',value = '1',

condition = c3$alcohol.user == 'former')

c3 <- add_col(data = c3,colname = 'alcohol.user2',value = '1',

condition = c3$alcohol.user == 'mild')

c3 <- add_col(data = c3,colname = 'alcohol.user2',value = '1',

condition = c3$alcohol.user == 'moderate')

c3 <- add_col(data = c3,colname = 'alcohol.user2',value = '1',

condition = c3$alcohol.user == 'heavy')

c3 <- add_col(data = c3,colname = 'alcohol.user2',value = '0',

condition = c3$alcohol.user == 'never')

c3$alcohol.user2 <- factor(c3$alcohol.user2, levels=c('0','1'))

#1.4吸烟 35211

c4 <- diag_smoke(years = 2009:2018)

c4 <- add_col(data = c4,colname = 'smoke2',value = '1',condition = c4$smoke == 'former')

c4 <- add_col(data = c4,colname = 'smoke2',value = '1',condition = c4$smoke == 'now')

c4 <- add_col(data = c4,colname = 'smoke2',value = '0',condition = c4$smoke == 'never')

c4$smoke2 <- factor(c4$smoke2, levels=c('0','1'))

#1.5高血压 48875

c5 <- diag_Hypertension(years = 2009:2018)

# Recode(c5$Hypertension)

c5$Hypertension <- Recode(c5$Hypertension,

"no::0",

"yes::1",

"NA::",

to.numeric = T)

c5$Hypertension <- factor(c5$Hypertension, levels=c('0','1'))

#1.6药物(高血压药) 49658

# c6 <- drug_anti.Hypertensive(years = 2009:2018,take_drug = TRUE,yes.code = 1,

# other.code = 0,no.code = 0,drugname = TRUE)

#

# c6 <- add_col(data = c6,colname = 'Drug2',value = 'NA',condition = c6$take_drug == 0)

# c6 <- add_col(data = c6,colname = 'Drug2',value = c6$Drug,condition = c6$take_drug == 1)

c6 <- drug_anti.Hypertensive(years = 2009:2018,take_drug = "antihp",yes.code = 1,

other.code = 0,no.code = 0,drugname = "hp_drug",dup.take.drug = "paste")

c6 <- add_col(data = c6,colname = 'RAS_Drug',value = '0',condition = c6$antihp == 0)

c6 <- add_col(data = c6,colname = 'RAS_Drug',value = c6$hp_drug,condition = c6$antihp == 1)

c6 <- add_col(data = c6,colname = 'RAS_Drug1',value = '0',condition = c6$antihp == 0)

c6 <- add_col(data = c6,colname = 'RAS_Drug1',value = "0",condition = c6$antihp == 1)

c6 <- add_col(data = c6,colname = 'RAS_Drug1',value = "1", condition = grepl("sartan$", c6$RAS_Drug)==TRUE

|grepl("pril$", c6$RAS_Drug)==TRUE |

grepl("sartan;", c6$RAS_Drug)==TRUE |

grepl("pril;", c6$RAS_Drug)==TRUE)

c6 <- drop_col(c6,'Drug')

#1.7 血常规 45744

c7 <- db_cbc(wbc_1000cells.ul = 'wbc',lymphocyte_number_1000cells.ul = 'lymphocyte',

Monocyte_number_1000cells.ul = 'monocyte',hemoglobin_g.dl = 'hgb',Platelet_count_1000cells.uL = 'plt'

,Segmented_neutrophils_number_1000cells.ul = 'neu', years = 2009:2018)

c7 <- add_col(data = c7,colname = 'SII',value = c7$plt*c7$neu/c7$lymphocyte)

c7 <- add_col(data = c7,colname = 'NLR',value = c7$neu/c7$lymphocyte)

c7 <- add_col(data = c7,colname = 'MLR',value = c7$monocyte/c7$lymphocyte)

c7 <- add_col(data = c7,colname = 'SIRI',value = c7$monocyte*c7$neu/c7$lymphocyte)

#1.8 尿蛋白 49379

c8 <- db_urine.alb.cr(uACR_mg.g = 'acr',years = 2009:2018)

c8 <- add_col(data = c8,colname = 'acr2',value = '<30',condition = c8$acr <30)

c8 <- add_col(data = c8,colname = 'acr2',value = '30≤acr<300',condition = c8$acr >= 30 & c8$acr <300)

c8 <- add_col(data = c8,colname = 'acr2',value = '≥300',condition = c8$acr >= 300)

# Recode(c8$acr2)

c8$acr2 <- Recode(c8$acr2,

"<30::0",

"30≤acr<300::1",

"≥300::2",

"NA::",

to.numeric = T)

c8$acr2 <- factor(c8$acr2, levels=c('0','1','2'))

#1.9 血生化 49491

c9 <- db_HemalBiochemistry(fast_glucose_mmol.L = 'fast_glu',refrige_glucose_mmol.L = 'refrige_glu',

HbA1c = "HbA1c",albumin_g.L = 'alb',

uric_acid_umol.L = 'uric_acid',calcium_total_mmol.L = "ca",

phosphorus_mmol.L = 'p',creatinine_umol.L = 'crea',potassium_mmol.L = 'k',

fast_triglyceride_mmol.L = 'fTG',refrige_total_cholesterol_mmol.L = 'TC',

hdl_cholesterol_mmol.L = 'HDL',ldl_cholesterol_mmol.L = 'LDL',

ldh_lactate_dehydrogenase_u.L = 'LDH',C_reactive_protein_mg.dl = 'CRP',

refrige_triglycerides_mmol.L = 'TG'

,follicle_stimulating_hormone_iu.L = 'follicle_stimulating_hormone',

luteinizing_hormone_iu.L = 'luteinizing_hormone',blood_urea_nitrogen_mmol.L = "BUN",

fast_total_cholesterol_mmol.L ='fTC', years = 2009:2018)

c9 <- add_col(data = c9,colname = 'alb2',value = '<35',condition = c9$alb <35)

c9 <- add_col(data = c9,colname = 'alb2',value = '>= 35',condition = c9$alb >= 35)

c9 <- add_col(data = c9,colname = 'HbA1c2',value = '<7.0', condition = c9$HbA1c <7.0)

c9 <- add_col(data = c9,colname = 'HbA1c2',value = '>= 7.0',condition = c9$HbA1c >= 7.0)

c9 <- add_col(data = c9,colname = 'HbA1c3',value = '<6.5', condition = c9$HbA1c <6.5)

c9 <- add_col(data = c9,colname = 'HbA1c3',value = '>= 6.5',condition = c9$HbA1c >= 6.5)

c9 <- add_col(data = c9,colname = 'TyG',value = log((c9$fTG*88.57)*(c9$fast_glu*18)/2))

# Recode(c9$alb2)

c9$alb2 <- Recode(c9$alb2,

">= 35::0",

"<35::1",

"NA::",

to.numeric = T)

c9$alb2 <- factor(c9$alb2, levels=c('0','1'))

#1.10 口服降糖药还是胰岛素 47715

tsv <- nhs_tsv(years = 2009:2018,'diq')

nhs_brief(tsv,'diq050')

x <- nhs_read(tsv,'diq050','diq070')

x1 <- add_col(data = x,colname = 'DM_drug',value = 'diabetic pills',condition = (x$diq070 == 'Yes'

& x$diq050 == 'No')| (x$diq070 == 'Yes' & x$diq050 == 'NA'))

x2 <- add_col(data = x1,colname = 'DM_drug',value = 'insulin',condition = (x$diq070 == 'No'

& x$diq050 == 'Yes')|(x$diq070 == 'NA' & x$diq050 == 'Yes'))

x3 <- add_col(data = x2,colname = 'DM_drug',value = 'diabetic pills_insulin',condition = x$diq070 == 'Yes'

& x$diq050 == 'Yes')

x4 <- add_col(data = x3,colname = 'DM_drug',value = 'no',condition = x$diq070 == 'No'

& x$diq050 == 'No')

c10 <- x4

c10 <- drop_col(c10,'Year','diq050','diq070')

# Recode(c10$DM_drug)

c10$DM_drug <- Recode(c10$DM_drug,

"diabetic pills::1",

"no::0",

"insulin::2",

"diabetic pills_insulin::3",

"NA::",

to.numeric = FALSE)

c10$DM_drug <- factor(c10$DM_drug, levels=c('0','1','2','3'))

# 1.11降糖药物分类处理

c11 <- drug_anti.Diabetic(years = 2009:2018,take_drug = T,drugname = T)

c11 <- add_col(data = c11,colname = 'DM_Drug2',value = "no",condition = c11$take_drug == "no")

c11 <- add_col(data = c11,colname = 'DM_Drug2',value = "NA",condition = c11$take_drug == "other")

c11 <- add_col(data = c11,colname = 'DM_Drug2',value = "yes", condition = c11$take_drug == "yes")

# Recode(a3$DM_Drug2)

c11$DM_Drug2 <- Recode(c11$DM_Drug2,

"glimepiride::sulfonylureas",

"insulin glargine::insulin",

"insulin zinc::insulin",

"metformin; pioglitazone::metformin_thiazolidinedione",

"glyburide::sulfonylureas",

"insulin::insulin",

"metformin::metformin",

"glipizide::sulfonylureas",

"insulin isophane::insulin",

"glyburide; metformin::metformin_sulfonylureas",

"insulin aspart::insulin",

"insulin lispro::insulin",

"antidiabetic agents - unspecified::other",

"repaglinide::glinides",

"insulin regular::insulin",

"pioglitazone::thiazolidinedione",

"insulin isophane; insulin regular::insulin",

"metformin; sitagliptin::metformin_DPP4",

"sitagliptin::DPP4",

"insulin glulisine::insulin",

"insulin lispro; insulin lispro protamine::insulin",

"chlorpropamide::sulfonylureas",

"insulin aspart; insulin aspart protamine::insulin",

"exenatide::GLP1",

"glipizide; metformin::metformin_sulfonylureas",

"metformin; rosiglitazone::metformin_thiazolidinedione",

"nateglinide::glinides",

"rosiglitazone::thiazolidinedione",

"acarbose::α_glucosidase_inhibitor",

"insulin detemir::insulin",

"metformin; saxagliptin::metformin_DPP4",

"linagliptin::DPP4",

"liraglutide::GLP1",

"gliclazide::sulfonylureas",

"insulin zinc extended::insulin",

"insulin isophane (nph)::insulin",

"saxagliptin::DPP4",

"linagliptin; metformin::metformin_DPP4",

"simvastatin; sitagliptin::DPP4",

"alogliptin; pioglitazone::thiazolidinedione",

"miglitol::α_glucosidase_inhibitor",

"dapagliflozin; metformin::metformin_SGLT2",

"canagliflozin; metformin::metformin_SGLT2",

"insulin degludec::insulin",

"empagliflozin; metformin::metformin_SGLT2",

"canagliflozin::SGLT2",

"dapagliflozin::SGLT2",

"NA::NA",

to.numeric = FALSE)

c11 <- drop_col(c11,'take_drug','Drug')

#1.12 诊断糖尿病，总人数47715，DM人数5606

c12 <- diag_DM(years = 2009:2018)

# Recode(c12)

c12$DM <- Recode(c12$DM,

"no::no",

"DM::yes",

"IGT::no",

"IFG::no",

"NA::",

to.numeric = F)

c12$DM <- factor(c12$DM, levels=c('no','yes'))

#1.13 诊断为CKD，总人数为49693，CKD人数：6608

c13 <- diag_CKD(years = 2009:2018, show_CKD = TRUE,

show_prognosis = TRUE, join = "left")

c13$CKD <- Recode(c13$CKD,

"no::0",

"yes::1", to.numeric = F)

c13$CKD <- as.character(c13$CKD)

c13$CKD <- factor(c13$CKD, levels = c("0", "1"))

#1.14 死亡数据

c14 <- db_mort(years = 2009:2018)

c14$mortstat <- Recode(c14$mortstat,'Assumed deceased::1','Assumed alive::0'

, to.numeric = TRUE)

c14 <- drop_col(c14,'eligstat','diabetes','hyperten')

col_rename(c14) <- c('mortstat:status','permth_exm:time','ucod_leading:leading')

# Recode(c14$leading)

c14$leading <- Recode(c14$leading,

"Diseases of heart (I00-I09, I11, I13, I20-I51)::CVD",

"Cerebrovascular diseases (I60-I69)::Cerebrovascular",

"All other causes (residual)::other",

"Chronic lower respiratory diseases (J40-J47)::lung",

"Malignant neoplasms (C00-C97)::cancer",

"Accidents (unintentional injuries) (V01-X59, Y85-Y86)::Accidents",

"Diabetes mellitus (E10-E14)::Diabetes",

"Alzheimer's disease (G30)::Alzheimer",

"Influenza and pneumonia (J09-J18)::",

"Nephritis, nephrotic syndrome and nephrosis (N00-N07, N17-N19, N25-N27)::kidney",

"NA::no",

to.numeric = FALSE)

summary(c14)

#1.15 加肾小球滤过率

c15 <- dex_eGFR(years = 2009:2018)

c15 <- add_col(data = c15, colname = 'e_GFR',value = "e_GFR<15", condition = c15$CKD_EPI_Scr_2009 < 15)

c15 <- add_col(data = c15, colname = 'e_GFR',value = "15≤e_GFR<30", condition = c15$CKD_EPI_Scr_2009 >= 15 & c15$CKD_EPI_Scr_2009 < 30)

c15 <- add_col(data = c15, colname = 'e_GFR',value = "30≤e_GFR<60", condition = c15$CKD_EPI_Scr_2009 >= 30 & c15$CKD_EPI_Scr_2009 < 60)

c15 <- add_col(data = c15, colname = 'e_GFR',value = "60≤e_GFR<90", condition = c15$CKD_EPI_Scr_2009 >= 60 & c15$CKD_EPI_Scr_2009 < 90)

c15 <- add_col(data = c15, colname = 'e_GFR',value = "e_GFR≥90", condition = c15$CKD_EPI_Scr_2009 >= 90)

c15 <- add_col(data = c15, colname = 'e_GFR2',value = "e_GFR<60", condition = c15$CKD_EPI_Scr_2009 < 60)

c15 <- add_col(data = c15, colname = 'e_GFR2',value = "e_GFR≥60", condition = c15$CKD_EPI_Scr_2009 >= 60)

c15 <- add_col(data = c15, colname = 'e_GFR3',value = "e_GFR<45", condition = c15$CKD_EPI_Scr_2009 < 45)

c15 <- add_col(data = c15, colname = 'e_GFR3',value = "e_GFR≥45", condition = c15$CKD_EPI_Scr_2009 >= 45)

# Recode(c15$e_GFR2)

c15$e_GFR2 <- Recode(c15$e_GFR2,

"e_GFR≥60::0",

"e_GFR<60::1",

"NA::",

to.numeric = T)

# Recode(c15$e_GFR)

c15$e_GFR <- Recode(c15$e_GFR,

"e_GFR≥90::0",

"60≤e_GFR<90::1",

"30≤e_GFR<60::2",

"15≤e_GFR<30::3",

"e_GFR<15::4",

"NA::",

to.numeric = T)

# Recode(c15$e_GFR3)

c15$e_GFR3 <- Recode(c15$e_GFR3,

"e_GFR≥45::0",

"e_GFR<45::1",

"NA::",

to.numeric = T)

#1.16 加是否是高脂血症

c16 <- diag_Hyperlipidemia(years = 2009:2018)

# Recode(c16$Hyperlipidemia)

c16$Hyperlipidemia <- Recode(c16$Hyperlipidemia,

"no::0",

"yes::1",

"NA::",

to.numeric = T)

c16$Hyperlipidemia <- factor(c16$Hyperlipidemia, levels=c('0','1'))

#1.17 加是不是贫血

c17 <- diag_Anemia(years = 2009:2018)

# Recode(c17)

c17$anemia <- Recode(c17$anemia,

"Non-Anaemia::0",

"Mild::1",

"age<0.5y::",

"Moderate::1",

"Severe::1",

"NA::",

to.numeric = T)

c17$anemia <- factor(c17$anemia, levels=c('0','1'))

# 1.18 加是不是CVD

c18 <- diag_CVD(years = 2009:2018)

# Recode(c18)

c18$CVD <- Recode(c18$CVD,

"no::0",

"yes::1",

"NA::",

to.numeric = T)

c18$CVD <- factor(c18$CVD, levels=c('0','1'))

###总表 n=49693

#1.19 教育程度

c19 <- db_demo(years = 2009:2018,edu = T)

# Recode(e$edu)

c19$edu <- Recode(c19$edu,

"High School Grad/GED or Equivalent::High_school_or_equivalent",

"8th Grade::Less_than_high_school",

"4th Grade::Less_than_high_school",

"9-11th Grade (Includes 12th grade with no diploma)::High_school_or_equivalent",

"Some College or AA degree::College_or_above",

"3rd Grade::Less_than_high_school",

"5th Grade::Less_than_high_school",

"10th Grade::High_school_or_equivalent",

"7th Grade::Less_than_high_school",

"Never Attended / Kindergarten Only::Less_than_high_school",

"2nd Grade::Less_than_high_school",

"College Graduate or above::College_or_above",

"Less Than 9th Grade::Less_than_high_school",

"High School Graduate::High_school_or_equivalent",

"1st Grade::Less_than_high_school",

"11th Grade::High_school_or_equivalent",

"6th Grade::Less_than_high_school",

"9th Grade::High_school_or_equivalent",

"More than high school::College_or_above",

"Less Than 5th Grade::Less_than_high_school",

"12th Grade, No Diploma::High_school_or_equivalent",

"GED or Equivalent::High_school_or_equivalent",

"High school graduate/GED or equivalent::High_school_or_equivalent",

"8th grade::Less_than_high_school",

"Some college or AA degree::College_or_above",

"7th grade::Less_than_high_school",

"3rd grade::Less_than_high_school",

"Never attended / kindergarten only::Less_than_high_school",

"9th grade::High_school_or_equivalent",

"College graduate or above::College_or_above",

"10th grade::High_school_or_equivalent",

"6th grade::Less_than_high_school",

"1st grade::Less_than_high_school",

"9-11th grade (Includes 12th grade with no diploma)::High_school_or_equivalent",

"2nd grade::Less_than_high_school",

"Less than 9th grade::Less_than_high_school",

"5th grade::Less_than_high_school",

"High school graduate::High_school_or_equivalent",

"4th grade::Less_than_high_school",

"11th grade::High_school_or_equivalent",

"12th grade, no diploma::High_school_or_equivalent",

"Less than 5th grade::Less_than_high_school",

"GED or equivalent::High_school_or_equivalent",

"NA::NA",

to.numeric = FALSE)

c19$edu <- factor(c19$edu, levels=c('Less_than_high_school','High_school_or_equivalent','College_or_above'))

#1.20 收入

c20 <- db_demo(years = 2009:2018,poverty = T,)

c20 <- add_col(data = c20,colname = 'poverty2',value = '0_1', condition = c20$poverty <1)

c20 <- add_col(data = c20,colname = 'poverty2',value = '1.1-3', condition = c20$poverty >= 1 & c20$poverty <3)

c20 <- add_col(data = c20,colname = 'poverty2',value = '>3', condition = c20$poverty >= 3)

c20$poverty2 <- factor(c20$poverty2, levels=c('0_1','1.1-3','>3'))

d1 <- db_drtot(years = 2009:2018,retinol_mcg = 'retinol',day = 1,fun = 'alone',energy_kcal = 'energy',

protein_g = 'protein',carbohydrate_g = 'carbohydrate',total_sugars_g = T,total_fat_g = 'fat',diet_lowfat = T,

diet_lowcarbohydrate = T,diet_kidney = T,diet_highprotein = T, wtdr2d = T,wtdrd1 = T)

#LCD评分

d1 <- add_col(data = d1, colname = 'carbohydrate_Score',value = "0", condition = (d1$carbohydrate*4)/d1$energy > 0.560)

d1 <- add_col(data = d1, colname = 'carbohydrate_Score',value = "1", condition = (d1$carbohydrate*4)/d1$energy > 0.516 &(d1$carbohydrate*4)/d1$energy <=0.560)

d1 <- add_col(data = d1, colname = 'carbohydrate_Score',value = "2", condition = (d1$carbohydrate*4)/d1$energy > 0.491 &(d1$carbohydrate*4)/d1$energy <=0.516)

d1 <- add_col(data = d1, colname = 'carbohydrate_Score',value = "3", condition = (d1$carbohydrate*4)/d1$energy > 0.471 &(d1$carbohydrate*4)/d1$energy <=0.491)

d1 <- add_col(data = d1, colname = 'carbohydrate_Score',value = "4", condition = (d1$carbohydrate*4)/d1$energy > 0.452 &(d1$carbohydrate*4)/d1$energy <=0.471)

d1 <- add_col(data = d1, colname = 'carbohydrate_Score',value = "5", condition = (d1$carbohydrate*4)/d1$energy > 0.433 &(d1$carbohydrate*4)/d1$energy <=0.452)

d1 <- add_col(data = d1, colname = 'carbohydrate_Score',value = "6", condition = (d1$carbohydrate*4)/d1$energy > 0.412 &(d1$carbohydrate*4)/d1$energy <=0.433)

d1 <- add_col(data = d1, colname = 'carbohydrate_Score',value = "7", condition = (d1$carbohydrate*4)/d1$energy > 0.388 &(d1$carbohydrate*4)/d1$energy <=0.412)

d1 <- add_col(data = d1, colname = 'carbohydrate_Score',value = "8", condition = (d1$carbohydrate*4)/d1$energy > 0.354 &(d1$carbohydrate*4)/d1$energy <=0.388)

d1 <- add_col(data = d1, colname = 'carbohydrate_Score',value = "9", condition = (d1$carbohydrate*4)/d1$energy > 0.293 &(d1$carbohydrate*4)/d1$energy <=0.354)

d1 <- add_col(data = d1, colname = 'carbohydrate_Score',value = "10", condition = (d1$carbohydrate*4)/d1$energy <=0.293)

d1$carbohydrate_Score <- as.numeric(d1$carbohydrate_Score )

d1 <- add_col(data = d1, colname = 'protein_Score',value = "0", condition = (d1$protein*4)/d1$energy < 0.141)

d1 <- add_col(data = d1, colname = 'protein_Score',value = "1", condition = (d1$protein*4)/d1$energy >=0.141 & (d1$protein*4)/d1$energy< 0.156)

d1 <- add_col(data = d1, colname = 'protein_Score',value = "2", condition = (d1$protein*4)/d1$energy >=0.156 & (d1$protein*4)/d1$energy< 0.166)

d1 <- add_col(data = d1, colname = 'protein_Score',value = "3", condition = (d1$protein*4)/d1$energy >=0.166 & (d1$protein*4)/d1$energy< 0.173)

d1 <- add_col(data = d1, colname = 'protein_Score',value = "4", condition = (d1$protein*4)/d1$energy >=0.173 & (d1$protein*4)/d1$energy< 0.180)

d1 <- add_col(data = d1, colname = 'protein_Score',value = "5", condition = (d1$protein*4)/d1$energy >=0.180 & (d1$protein*4)/d1$energy< 0.187)

d1 <- add_col(data = d1, colname = 'protein_Score',value = "6", condition = (d1$protein*4)/d1$energy >=0.187 & (d1$protein*4)/d1$energy< 0.194)

d1 <- add_col(data = d1, colname = 'protein_Score',value = "7", condition = (d1$protein*4)/d1$energy >=0.194 & (d1$protein*4)/d1$energy< 0.203)

d1 <- add_col(data = d1, colname = 'protein_Score',value = "8", condition = (d1$protein*4)/d1$energy >=0.203 & (d1$protein*4)/d1$energy< 0.215)

d1 <- add_col(data = d1, colname = 'protein_Score',value = "9", condition = (d1$protein*4)/d1$energy >=0.215 & (d1$protein*4)/d1$energy< 0.240)

d1 <- add_col(data = d1, colname = 'protein_Score',value = "10", condition = (d1$protein*4)/d1$energy >=0.240)

d1$protein_Score <- as.numeric(d1$protein_Score )

d1 <- add_col(data = d1, colname = 'fat_Score',value = "0", condition = (d1$fat*9)/d1$energy <0.260)

d1 <- add_col(data = d1, colname = 'fat_Score',value = "1", condition = (d1$fat*9)/d1$energy >=0.260 & (d1$fat*9)/d1$energy<0.295)

d1 <- add_col(data = d1, colname = 'fat_Score',value = "2", condition = (d1$fat*9)/d1$energy >=0.295 & (d1$fat*9)/d1$energy<0.316)

d1 <- add_col(data = d1, colname = 'fat_Score',value = "3", condition = (d1$fat*9)/d1$energy >=0.316 & (d1$fat*9)/d1$energy<0.332)

d1 <- add_col(data = d1, colname = 'fat_Score',value = "4", condition = (d1$fat*9)/d1$energy >=0.332 & (d1$fat*9)/d1$energy<0.347)

d1 <- add_col(data = d1, colname = 'fat_Score',value = "5", condition = (d1$fat*9)/d1$energy >=0.347 & (d1$fat*9)/d1$energy<0.361)

d1 <- add_col(data = d1, colname = 'fat_Score',value = "6", condition = (d1$fat*9)/d1$energy >=0.361 & (d1$fat*9)/d1$energy<0.377)

d1 <- add_col(data = d1, colname = 'fat_Score',value = "7", condition = (d1$fat*9)/d1$energy >=0.377 & (d1$fat*9)/d1$energy<0.395)

d1 <- add_col(data = d1, colname = 'fat_Score',value = "8", condition = (d1$fat*9)/d1$energy >=0.395 & (d1$fat*9)/d1$energy<0.420)

d1 <- add_col(data = d1, colname = 'fat_Score',value = "9", condition = (d1$fat*9)/d1$energy >=0.420 & (d1$fat*9)/d1$energy<0.469)

d1 <- add_col(data = d1, colname = 'fat_Score',value = "10", condition = (d1$fat*9)/d1$energy >=0.469)

d1$fat_Score <- as.numeric(d1$fat_Score )

d1 <- add_col(data = d1, colname = 'Score_all',value = d1$carbohydrate_Score + d1$protein_Score + d1$fat_Score)

d2 <- diag_PHQ9(

years= 2009:2018,

na0 = T,

score = T,

dpq = F,

varLabel = F,

cat = T)

C <- Left_Join(c1,c2,c3,c4,c5,c6,c7,c8,c9,c10,c11,c12,c13,c14,c15,c16,c17,c18,c19,c20,d1,d2)

#________________________________________________________2.排除

#1.排除小于18岁的 49693-20858 28835

C <- select_row(C,C$age >= 20)

#2.怀孕 28835-316

p1 <- diag_Pregnant(years = 2009:2018)

C <- Left_Join(C, p1)

C <- drop_row(C,C$Pregnant == 'yes')

#3.排除不是糖尿病以及1型糖尿病 30036-24507 5529-122 5407

C1 <- select_row(C,C$DM =='yes')

diq<-nhs_tsv('diq',years = 2009:2018)

c24<-nhs_read(diq,

'DID040:DMage',

'DIQ050:insulinuse',

'DIQ070:DMpills',lower_cd = TRUE)

C2 <- Left_Join(C1,c24)

ck <- C2$DMage <= 30 & C2$insulinuse == 'yes' & C2$DMpills == 'no'

C3 <- select_row(C2,!ck)

# group1 + group2 4888

D1 <- select_row(C3,C3$CKD =='0' | C3$CKD =='1')

# # # ###排除透析

# diq<-nhs_tsv('kiq',years = 2009:2018)

# c24<-nhs_read(diq, 'kiq025:dialysis',lower_cd = TRUE) ###108

#

# D1 <- Left_Join(D1, c24)

# D1 <- drop_row(D1,D1$dialysis == 'yes') ###28411

D1<- drop_row(D1,is.na(D1$CKD))

D1$CKD <- Recode(D1$CKD,

"no::0",

"yes::1",

"NA::",

to.numeric = T)

D1<- drop_row(D1,is.na(D1$Score_all))

str(D1)

# #___________________________________________插补

# summary(D1)

#

D1 <- drop_col(D1,'height','alcohol.user','smoke','take_drug','Drug2','wbc','lymphocyte','monocyte',

'neu','plt','BUN','p','ca','k','LDH',

'CKD_prognosis','Pregnant','Year.y','diet_kidney','total_sugars_g',

'retinol','time','status','permth_int','leading','crea',

"dialysis",'wtdr2d','diet_lowfat','diet_lowcarbohydrate','diet_highprotein')

#

# View(C)

#

# # 定义需要选择的变量列表

selected_vars <- c('Score_all', 'age', 'HbA1c', 'acr', 'CKD_EPI_Scr_2009',

'sex', 'ethic', 'BMI', 'alcohol.user2', 'smoke2', 'edu',

'poverty2', 'Hypertension', 'Hyperlipidemia', 'DM_drug',

'Year.x')

#

# ——————————————————————————————————查看缺失

library(mice)

md.pattern(D1)

summary(D1)

# ——————————————————————————————————缺失比例及可视化

library(VIM)

aggr_plot <- aggr(D1, col=c('navyblue','red'),

numbers=TRUE, sortVars=TRUE, labels=names(D1),

cex.axis=.7, gap=3, ylab=c("Histogram of missing data","Pattern"))

# ————————————————————————————————————插补

tempData <- mice(D1,m=5,maxit=10,meth='pmm',seed=100)

summary(tempData)

# ————————————————————————————————————插补的第一个数据集

D1 <- complete(tempData,2)

# 设置更大的图形设备

png("缺失模式分析.png", width = 2000, height = 3000, res = 150)

VIM::aggr(D1, col = c('navyblue','red'), numbers = TRUE, sortVars = TRUE)

dev.off()

cat("缺失模式图已保存为'缺失模式分析.png'，请查看该文件\n")

str(D1)

# 检查默认插补方法

init <- mice(D1, maxit =0)# maxit=0表示只初始化，不进行插补

meth <- init$method

print("默认插补方法：")

print(meth)

# 根据变量类型优化插补方法

meth["Score_all"]<-"pmm"# 预测均值匹配（适合连续变量）

meth["age"]<-"pmm"# 预测均值匹配

meth["HbA1c"]<-"pmm"# 预测均值匹配

meth["acr"]<-"pmm"# 预测均值匹配

meth["CKD_EPI_Scr_2009"]<-"pmm"# 预测均值匹配

# meth["energy"]<-"pmm"# 预测均值匹配

meth["sex"]<-"logreg"# 逻辑回归（适合二分类变量

meth["smoke2"]<-"logreg"# 逻辑回归（适合二分类变量

meth["alcohol.user2"]<-"logreg"# 逻辑回归（适合二分类变量

meth["Hypertension"]<-"logreg"# 逻辑回归（适合二分类变量

meth["Hyperlipidemia"]<-"logreg"# 逻辑回归（适合二分类变量

meth["ethic"]<-"polyreg"# 多元逻辑回归（适合多分类变量）

meth["BMI2"]<-"polyreg"# 多元逻辑回归（适合多分类变量）

meth["edu"]<-"polyreg"# 多元逻辑回归（适合多分类变量）

meth["Year.x"]<-"polyreg"# 多元逻辑回归（适合多分类变量）

meth["DM_drug"]<-"polyreg"# 多元逻辑回归（适合多分类变量）

meth["poverty2"]<-"polyreg"# 多元逻辑回归（适合多分类变量

set.seed(123)

# 比较不同插补次数的效果

imp_5 <- mice (D1, method = meth, m =5, maxit =10, print =FALSE, seed =123)

imp_10 <- mice(D1, method = meth, m =10, maxit =10, print =FALSE, seed =123)

imp_20 <- mice(D1, method = meth, m =20, maxit =10, print =FALSE, seed =123)

# 收敛性诊断

print("插补收敛诊断：")

plot(imp_10, main ="Convergence Diagnostics")

# 检查插补值的分布

stripplot(imp_10, age ~ .imp, pch =20, cex =1.2)# 条带图

densityplot(imp_10,~age, main ="Distribution of Age")# 密度图

# 直接修复您当前的代码

missing_vars <- names(imp_10$imp)

cat("需要诊断的变量数量:", length(missing_vars), "\n")

# 只处理确实在D1中存在的变量

valid_vars <- missing_vars[missing_vars %in% names(D1)]

cat("实际可诊断的变量:", paste(valid_vars, collapse = ", "), "\n")

# 分批处理

for(i in seq(1, length(valid_vars), by = 3)) {

batch <- valid_vars[i:min(i+2, length(valid_vars))]

png(paste0("收敛诊断_批次", ceiling(i/3), ".png"), width = 1200, height = 800)

plot(imp_10, batch, main = paste("收敛诊断 - 批次", ceiling(i/3)))

dev.off()

cat("已生成批次", ceiling(i/3), "的收敛诊断图\n")

}

D1 <- D1_data

library(mice)

library(ggplot2)

library(gridExtra)

library(broom)

library(coda)

# 创建输出目录

output_dir <- "收敛性诊断与比较分析"

if (!dir.exists(output_dir)) {

dir.create(output_dir)

}

# 定义变量列表（根据您的说明）

continuous_vars <- c("HbA1c", "acr", "CKD_EPI_Scr_2009") # 数值变量

categorical_vars <- c("DM_drug", "poverty2", "alcohol.user2",

"BMI2", "acr2", "edu", "smoke2") # 分类变量

# 合并所有变量

all_vars <- c(continuous_vars, categorical_vars)

# 验证变量是否存在於数据框中

existing_vars <- all_vars[all_vars %in% names(D1)]

cat("将分析的变量:", paste(existing_vars, collapse = ", "), "\n")

# 重新运行插补，确保正确记录分类变量

# tempData <- mice(D1, m = 5, maxit = 10, meth = 'pmm', seed = 100)

tempData <- mice (D1, method = meth, m =5, maxit =10, seed =123)

# 检查D1的缺失值模式

missing_pattern <- md.pattern(D1, plot = TRUE)

print(missing_pattern)

# 在运行诊断前验证数据

cat("=== 数据预处理验证 ===\n")

for(var in categorical_vars) {

if(var %in% names(D1)) {

cat(sprintf("变量 %s: 类型=%s, 类别数=%d, 缺失值=%d\n",

var,

class(D1[[var]]),

length(unique(na.omit(D1[[var]]))),

sum(is.na(D1[[var]]))))

}

}

# 加载必要的包

library(coda)

# 替换原有的Gelman-Rubin计算部分

tryCatch({

# 正确的方法是将mids对象转换为mcmc列表

mcmc_list <- lapply(1:tempData$m, function(i) {

chain_data <- mice::complete(tempData, i)

# 提取连续变量并转换为mcmc对象

as.mcmc(chain_data[, continuous_vars])

})

# 将列表转换为mcmc.list

mcmc_list <- as.mcmc.list(mcmc_list)

# 计算Gelman-Rubin统计量

gr_stats <- gelman.diag(mcmc_list, multivariate = FALSE)

cat("\nGelman-Rubin统计量 (应接近1.0):\n")

print(gr_stats$psrf)

# 检查收敛性

converged_vars <- names(which(gr_stats$psrf[, "Point est."] < 1.1))

not_converged_vars <- names(which(gr_stats$psrf[, "Point est."] >= 1.1))

cat("\n收敛变量:", paste(converged_vars, collapse = ", "), "\n")

if(length(not_converged_vars) > 0) {

cat("未完全收敛变量:", paste(not_converged_vars, collapse = ", "), "\n")

cat("建议增加迭代次数或检查插补模型\n")

}

}, error = function(e) {

cat("Gelman-Rubin计算错误:", e$message, "\n")

})

# 设置图形参数

par(mfrow = c(2, 3)) # 2行3列，容纳6个子图

# 绘制三个变量的轨迹图

plot(tempData, vars = c("HbA1c", "acr", "CKD_EPI_Scr_2009"),

main = "关键变量轨迹图")

# 或者分别绘制每个变量

plot(tempData, vars = "HbA1c", main = "HbA1c")

plot(tempData, vars = "acr", main = "acr")

plot(tempData, vars = "CKD_EPI_Scr_2009", main = "CKD_EPI_Scr_2009")

# 步骤1：重新配置插补方法

# 1. 首先定义分类变量列表（替换为您的实际变量名）

categorical_vars <- c("DM_drug", "poverty2", "alcohol.user2", "BMI2", "acr2", "edu", "smoke2")

# 2. 初始化插补方法向量

# 方法1：使用mice包的make.method函数

imp_methods <- mice::make.method(D1)

# 方法2：手动创建空向量

# imp_methods <- character(length(names(D1)))

# names(imp_methods) <- names(D1)

# 3. 根据变量类型设置不同方法（修正版）

for(var in categorical_vars) {

if(var %in% names(D1)) {

if(is.factor(D1[[var]]) || is.character(D1[[var]])) {

# 获取类别数量（添加错误处理）

tryCatch({

n_categories <- length(unique(na.omit(D1[[var]])))

cat("变量", var, "有", n_categories, "个类别\n")

if(n_categories == 2) {

imp_methods[var] <- "logreg" # 二分类逻辑回归

cat(" 设置为logreg方法\n")

} else if(n_categories > 2) {

imp_methods[var] <- "polyreg" # 多分类多项式回归

cat(" 设置为polyreg方法\n")

} else if(n_categories == 1) {

warning("变量", var, "只有1个类别，无法插补")

imp_methods[var] <- "" # 跳过插补

}

}, error = function(e) {

cat("处理变量", var, "时出错:", e$message, "\n")

})

} else {

cat("变量", var, "不是因子或字符型，当前类型:", class(D1[[var]]), "\n")

}

} else {

cat("变量", var, "不在数据框D1中\n")

}

}

# 4. 检查最终配置

cat("\n=== 最终插补方法配置 ===\n")

for(var in categorical_vars) {

if(var %in% names(imp_methods)) {

cat(var, ":", imp_methods[var], "\n")

}

}

# 为连续变量保留PMM方法

continuous_vars <- c("HbA1c", "acr", "CKD_EPI_Scr_2009")

for(var in continuous_vars) {

if(var %in% names(D1)) {

imp_methods[var] <- "pmm"

}

}

# 查看最终的方法设置

print("插补方法配置:")

print(imp_methods[imp_methods != ""])

# 重新运行插补

tempData_corrected <- mice(D1, m = 5, maxit = 10,

method = imp_methods,

seed = 100, # 可考虑移除或更改种子以增加随机性

printFlag = TRUE)

# 步骤3：验证修正效果

# 完整的收敛性诊断函数（修正版）

run_convergence_diagnostics <- function(tempData, continuous_vars, categorical_vars, output_dir) {

# 确保输出目录存在

if (!dir.exists(output_dir)) {

dir.create(output_dir, recursive = TRUE)

cat("已创建输出目录:", output_dir, "\n")

}

# 创建诊断报告文件

report_file <- file.path(output_dir, "convergence_diagnostics_report.txt")

sink(report_file)

cat("=== 多重插补收敛性诊断报告 ===\n")

cat("生成时间:", format(Sys.time(), "%Y-%m-%d %H:%M:%S"), "\n\n")

# 连续变量的收敛性诊断

if(length(continuous_vars) > 0) {

cat("--- 连续变量收敛性诊断 ---\n")

# 生成轨迹图

png(file.path(output_dir, "continuous_vars_trace_plots.png"),

width = 1200, height = 800, res = 150)

plot(tempData, layout = c(2, 2), main = "连续变量插补轨迹图")

dev.off()

cat("连续变量轨迹图已保存: continuous_vars_trace_plots.png\n")

# 计算Gelman-Rubin统计量

tryCatch({

# 使用coda包的正确方法

library(coda)

mcmc_list <- lapply(1:tempData$m, function(i) {

chain_data <- mice::complete(tempData, i)

as.mcmc(chain_data[, continuous_vars, drop = FALSE])

})

mcmc_list <- as.mcmc.list(mcmc_list)

gr_stats <- gelman.diag(mcmc_list, multivariate = FALSE)

cat("\nGelman-Rubin统计量 (应接近1.0):\n")

print(gr_stats$psrf)

# 检查收敛性

converged_vars <- names(which(gr_stats$psrf[, "Point est."] < 1.1))

not_converged_vars <- names(which(gr_stats$psrf[, "Point est."] >= 1.1))

cat("\n收敛变量:", paste(converged_vars, collapse = ", "), "\n")

if(length(not_converged_vars) > 0) {

cat("未完全收敛变量:", paste(not_converged_vars, collapse = ", "), "\n")

cat("建议增加迭代次数或检查插补模型\n")

}

}, error = function(e) {

cat("Gelman-Rubin计算错误:", e$message, "\n")

})

}

# 分类变量的收敛性诊断

if(length(categorical_vars) > 0) {

cat("\n--- 分类变量收敛性诊断 ---\n")

for(var in categorical_vars) {

if(var %in% names(D1)) {

cat("\n处理变量:", var, "\n")

tryCatch({

# 获取类别水平

if(is.factor(D1[[var]])) {

categories <- levels(D1[[var]])

} else {

categories <- unique(na.omit(D1[[var]]))

}

if(length(categories) == 0) {

warning("变量 ", var, " 没有有效的类别水平，跳过处理")

next

}

cat("检测到类别:", paste(categories, collapse = ", "), "\n")

# 初始化比例矩阵

chain_proportions <- matrix(NA, nrow = tempData$m, ncol = length(categories))

colnames(chain_proportions) <- categories

# 计算每个链的比例

for(chain in 1:tempData$m) {

completed_data <- mice::complete(tempData, chain)

tab <- table(completed_data[[var]])

proportions <- prop.table(tab)

for(i in seq_along(categories)) {

category_name <- categories[i]

if(category_name %in% names(proportions)) {

chain_proportions[chain, i] <- proportions[category_name]

} else {

chain_proportions[chain, i] <- 0

}

}

}

# 计算变异系数(CV)

cv_values <- apply(chain_proportions, 2, function(x) {

if(mean(x, na.rm = TRUE) > 0) {

sd(x, na.rm = TRUE) / mean(x, na.rm = TRUE) * 100

} else {

NA

}

})

avg_cv <- mean(cv_values, na.rm = TRUE)

cat("平均变异系数(CV):", round(avg_cv, 2), "%\n")

# 保存比例稳定性图

png(file.path(output_dir, paste0("proportion_stability_", var, ".png")),

width = 800, height = 600, res = 150)

matplot(1:tempData$m, chain_proportions, type = "b", pch = 16, lwd = 2,

main = paste("比例稳定性:", var),

xlab = "链编号", ylab = "类别比例",

xlim = c(0.5, tempData$m + 0.5), ylim = c(0, 1),

col = 1:length(categories), lty = 1)

legend("topright", legend = categories,

col = 1:length(categories), pch = 16, cex = 0.7)

grid()

mtext(sprintf("平均CV=%.2f%%", avg_cv), side = 3, line = 0, cex = 0.8)

dev.off()

}, error = function(e) {

warning("处理变量 ", var, " 时出错: ", e$message)

})

}

}

}

sink()

cat("诊断报告已保存至:", report_file, "\n")

}

# 使用示例

# 确保目录存在

if (!dir.exists("最终分析结果")) {

dir.create("最终分析结果", recursive = TRUE)

}

# 运行诊断

run_convergence_diagnostics(tempData_final, continuous_vars, categorical_vars, "最终分析结果")

# 确保输出目录存在

if (!dir.exists("最终分析结果")) {

dir.create("最终分析结果", recursive = TRUE)

cat("已创建输出目录: 最终分析结果\n")

}

# 然后运行诊断函数

run_convergence_diagnostics(tempData_final, continuous_vars, categorical_vars, "最终分析结果")

D1 <- add_col(data = D1, colname = 'energy2',value = D1$energy)

D1 <- add_col(data = D1, colname = 'energy3',value = D1$energy)

D1$energy2 <- quant( D1$energy2, n=2, Q = TRUE, round = 3)

D1$energy2 <- factor(D1$energy2, levels=c('Q1','Q2'))

D1$energy3 <- quant( D1$energy3, n=3, Q = TRUE, round = 3)

D1$energy3 <- factor(D1$energy3, levels=c('Q1','Q2','Q3'))

D1 <- add_col(data = D1, colname = 'energy4',value = D1$Score_all/D1$energy)

D1 <- add_col(data = D1, colname = 'energy5',value = D1$Score_all/D1$energy)

D1$energy4 <- quant( D1$energy4, n=2, Q = TRUE, round = 3)

D1$energy4 <- factor(D1$energy4, levels=c('Q1','Q2'))

D1$energy5 <- quant( D1$energy5, n=5, Q = TRUE, round = 3)

D1$energy5 <- factor(D1$energy5, levels=c('Q1','Q2','Q3','Q4',"Q5"))

D1 <- add_col(data = D1, colname = 'Score_all2',value = D1$Score_all)

D1 <- add_col(data = D1, colname = 'Score_all3',value = D1$Score_all)

D1 <- add_col(data = D1, colname = 'Score_all4',value = D1$Score_all)

D1$Score_all2 <- quant( D1$Score_all2, n=2, Q = TRUE, round = 3)

D1$Score_all2 <- factor(D1$Score_all2, levels=c('Q1','Q2'))

D1$Score_all3 <- quant( D1$Score_all3, n=3, Q = TRUE, round = 3)

# D1$energy4 <- factor(D1$energy4, levels=c('Q3','Q2','Q1'))

D1$Score_all3 <- factor(D1$Score_all3, levels=c('Q1','Q2','Q3'))

D1$Score_all4 <- quant( D1$Score_all4, n=4, Q = TRUE, round = 3)

# D1$energy5 <- factor(D1$energy5, levels=c('Q1','Q2','Q3','Q4'))

D1$Score_all4 <- factor(D1$Score_all4, levels=c('Q1','Q2','Q3','Q4'))

D1$Score_all.scaled <- scale(D1$Score_all)

# # 加载必要的包

library(readxl)

library(writexl)

# # 设置随机种子保证结果可重复

# set.seed(123)

#

# # 从D1中随机抽取37例患者

# D2 <- D1[sample(nrow(D1), 37), ]

#

# # 将结果写入Excel到桌面

write_xlsx(D1, "E:/桌面/D4.xlsx")

#________________________________________________________3.加权

D1 <- drop_row(D1,is.na(D1$wtdrd1))

D1 <- drop_row(D1,D1$wtdrd1 == 0)

D1$nhs_wt <- 1/5 * D1$wtdrd1

D1 <- drop_col(D1, "wtdrd1")

nhs <- svy_design(data = D1)

svy_population(design = nhs)

#________________________________________________________4.分析

library(nhanesR)

library(survey)

library(rms)

library(tableone)

library(readxl)

library(dplyr)

# 读取数据

D2 <- read.csv("D2.csv") # 插补前数据

D3 <- read.csv("D3.csv") # 插补后数据

# 确保seqn为字符型，避免格式问题

D2$seqn <- as.character(D2$seqn)

D3$seqn <- as.character(D3$seqn)

# 指定要分析的变量

variables <- c("acr", "CKD_EPI_Scr_2009", "BMI2", "ethic", "sex",

"alcohol.user2", "smoke2", "edu", "poverty2", "Hypertension",

"Hyperlipidemia", "DM_drug", "e_GFR2", "acr2")

# 指定分类变量

categorical_vars <- c("ethic", "sex", "alcohol.user2", "smoke2", "edu",

"poverty2", "Hypertension", "Hyperlipidemia", "DM_drug")

# 创建分组变量：Before (D2) 和 After (D3) imputation

D2$group <- "Before"

D3$group <- "After"

# 合并两个数据集

combined_data <- bind_rows(D2, D3)

combined_data$group <- factor(combined_data$group)

# 使用tableone包创建基线特征表并计算SMD

table_smd <- CreateTableOne(vars = variables,

strata = "group",

data = combined_data,

factorVars = categorical_vars,

smd = TRUE)

# 输出结果

print(table_smd, smd = TRUE, showAllLevels = TRUE)

# 提取SMD值

smd_results <- ExtractSmd(table_smd)

print(smd_results)

# 创建SMD结果的数据框

smd_df <- data.frame(

Variable = names(smd_results),

SMD = round(smd_results, 4)

)

# 打印美观的结果表

print(smd_df)

# 可视化SMD结果

plot(smd_df$SMD, type = "h", lwd = 3, col = ifelse(abs(smd_df$SMD) > 0.1, "red", "blue"),

xlab = "Variable Index", ylab = "Standardized Mean Difference (SMD)",

main = "SMD Before vs After Multiple Imputation")

abline(h = c(-0.1, 0.1), lty = 2, col = "gray")

text(x = 1:nrow(smd_df), y = smd_df$SMD, labels = smd_df$Variable,

pos = 3, cex = 0.7, srt = 45)

legend("topright", legend = c("|SMD| > 0.1", "|SMD| ≤ 0.1"),

fill = c("red", "blue"), bty = "n")

# 加载必要的包

library(dplyr)

library(tidyr)

# 检查数据是否存在

if (!exists("D1")) {

# 如果D1不存在，尝试从文件读取

if (file.exists("D1.csv")) {

D1 <- read.csv("D1.csv", stringsAsFactors = FALSE)

cat("从D1.csv读取数据\n")

} else if (file.exists("D1.xlsx")) {

library(readxl)

D1 <- read_excel("D1.xlsx")

cat("从D1.xlsx读取数据\n")

} else if (file.exists("D1.rds")) {

D1 <- readRDS("D1.rds")

cat("从D1.rds读取数据\n")

} else {

stop("找不到D1数据。请确保数据已加载或文件存在。")

}

}

# 检查必要的列是否存在

required_cols <- c("Score_all", "nhs_wt")

missing_cols <- setdiff(required_cols, names(D1))

if (length(missing_cols) > 0) {

stop(paste("以下列在数据中不存在:", paste(missing_cols, collapse = ", "),

"\n数据中的列名:", paste(names(D1), collapse = ", ")))

}

cat("数据维度:", dim(D1), "(行数, 列数)\n")

# 定义区间（与图片完全一致）

intervals <- c("< 10", "10 - 15", "15 - 20", "20 - 25", "> 25")

# 计算频数分布和加权百分比

calculate_weighted_distribution <- function(data) {

# 创建区间变量

data_with_interval <- data %>%

mutate(

Score_Interval = case_when(

Score_all < 10 ~ "< 10",

Score_all >= 10 & Score_all < 15 ~ "10 - 15",

Score_all >= 15 & Score_all < 20 ~ "15 - 20",

Score_all >= 20 & Score_all <= 25 ~ "20 - 25", # 包括25

Score_all > 25 ~ "> 25",

TRUE ~ NA_character_

)

) %>%

mutate(Score_Interval = factor(Score_Interval, levels = intervals)) %>%

filter(!is.na(Score_Interval)) # 排除缺失值

# 计算每个区间的统计量

result <- data_with_interval %>%

group_by(Score_Interval) %>%

summarise(

n = n(), # 实际观测数

weighted_sum = sum(nhs_wt, na.rm = TRUE) # 加权总和

) %>%

ungroup()

# 计算加权百分比

total_weighted <- sum(result$weighted_sum, na.rm = TRUE)

result$`Weighted %` <- round(result$weighted_sum / total_weighted * 100, 1)

return(result)

}

# 计算结果

result <- calculate_weighted_distribution(D1)

# 添加总计行

total_row <- data.frame(

Score_Interval = "Total",

n = nrow(D1[!is.na(D1$Score_all), ]), # 排除Score_all为NA的行

weighted_sum = sum(D1$nhs_wt, na.rm = TRUE),

`Weighted %` = 100.0

)

names(total_row) <- names(result)

final_result <- rbind(result, total_row)

# 美化列名以匹配图片格式

colnames(final_result) <- c("LCDS Score Interval", "n", "Weighted Sum", "Weighted %")

# 重新格式化输出

final_result_formatted <- data.frame(

"LCDS Score Interval" = final_result$`LCDS Score Interval`,

"n" = final_result$n,

"Weighted %" = ifelse(is.na(final_result$`Weighted %`),

"",

sprintf("%.1f%%", final_result$`Weighted %`))

)

# 打印结果

cat("\n=== LCDS Score 分布表 ===\n")

print(final_result_formatted, row.names = FALSE)

f0 <- svyglm(CKD~ rcs(Score_all,3), design = nhs, family = quasibinomial())

#参考点

optimal_nKnots(f0)

#参考点

f0 <- RCS(f0)

ggplot(f0,mapping = F,xlab = 'LCD_score)')

#获取拐点

getChangepoints(f0)

# 加载必要的包

library(ggplot2)

library(patchwork)

library(survey) # 确保加载survey包，它提供了svyglm和对应的predict方法

# 假设您的复杂抽样设计对象 'nhs' 和模型 'f0' 已经正确创建

# 加载必要的包

library(ggplot2)

library(patchwork)

library(survey)

# 1. 重新拟合模型（确保f0是有效的svyglm对象）

f0 <- svyglm(CKD ~ rcs(Score_all, 3), design = nhs, family = quasibinomial())

# 2. 生成预测数据框

pred_data <- data.frame(

Score_all = seq(

from = min(nhs$variables$Score_all, na.rm = TRUE),

to = max(nhs$variables$Score_all, na.rm = TRUE),

length.out = 100

)

)

# 3. 进行预测并正确处理预测结果

pred_values <- predict(f0, newdata = pred_data, type = "response")

# 4. 诊断pred_values的结构[7](@ref)

print("pred_values的结构:")

print(str(pred_values))

print("pred_values的类别:")

print(class(pred_values))

# 5. 根据pred_values的实际结构创建plot_data

# 方法1：如果pred_values是数值向量

plot_data <- data.frame(Score_all = pred_data$Score_all,

Predicted_Prob = as.numeric(pred_values))

# 方法2：如果pred_values是矩阵或数组，提取第一列

# plot_data <- data.frame(Score_all = pred_data$Score_all,

# Predicted_Prob = as.numeric(pred_values[,1]))

# 6. 检查plot_data的最终结构

print("plot_data的列名:")

print(names(plot_data))

print("plot_data的前几行:")

print(head(plot_data))

# 7. 绘制RCS曲线主图（修正版）

p_main <- ggplot(data = plot_data, aes(x = Score_all, y = Predicted_Prob)) +

geom_line(color = "red", linewidth = 1) +

labs(x = "LCD_score", y = "Predicted Probability of CKD") +

theme_bw()

# 8. 绘制Score_all的分布直方图

p_hist <- ggplot(nhs$variables, aes(x = Score_all)) +

geom_histogram(fill = "skyblue", color = "black", alpha = 0.7, bins = 30) +

labs(x = "LCD_score", y = "Frequency") +

theme_bw()

# 9. 组合图形

combined_plot <- p_main / p_hist +

plot_layout(heights = c(3, 1))

# 10. 显示图形

print(combined_plot)

# 1. 加载包并确保成功

library(survey)

library(ggplot2)

library(patchwork)

# 确保rms包已安装和加载

if (!requireNamespace("rms", quietly = TRUE)) {

install.packages("rms")

}

library(rms)

# 2. 检查数据对象和变量

print("=== 开始检查数据 ===")

if (!exists("nhs")) {

stop("错误：未找到名为 'nhs' 的数据对象。请检查对象名是否正确。")

}

# 检查变量

if (!all(c("CKD", "Score_all") %in% names(nhs$variables))) {

stop("错误：在 'nhs$variables' 中未找到 'CKD' 或 'Score_all' 变量。")

}

# 3. 设置rms包所需的数据环境（这步很重要！）

# 注意：由于nhs是survey.design对象，我们需要对其中的variables进行操作

ddist <- datadist(nhs$variables)

options(datadist = "ddist")

# 4. 重新拟合模型

print("=== 开始拟合模型 ===")

tryCatch({

# 使用tryCatch捕获错误

f0 <- svyglm(CKD ~ rcs(Score_all, 3), design = nhs, family = quasibinomial())

print("模型拟合成功！")

print(summary(f0))

}, error = function(e) {

print(paste("模型拟合出错:", e$message))

})

# 如果模型拟合成功，再继续预测和绘图

if (exists("f0")) {

print("=== 开始预测 ===")

# 5. 创建预测数据

pred_data <- data.frame(

Score_all = seq(

from = min(nhs$variables$Score_all, na.rm = TRUE),

to = max(nhs$variables$Score_all, na.rm = TRUE),

length.out = 100

)

)

# 6. 进行预测 - 尝试两种方式

tryCatch({

# 方式1：使用survey包的predict方法

pred_values <- predict(f0, newdata = pred_data, type = "response")

# 通常predict返回一个向量或矩阵，我们将其转换为数据框

plot_data <- data.frame(Score_all = pred_data$Score_all,

Predicted_Prob = as.numeric(pred_values))

print("预测成功！")

}, error = function(e) {

print(paste("预测出错:", e$message))

})

# 7. 绘图

if (exists("plot_data")) {

print("=== 开始绘图 ===")

p_main <- ggplot(plot_data, aes(x = Score_all, y = Predicted_Prob)) +

geom_line(color = "red", linewidth = 1) +

labs(x = "LCD_score", y = "Predicted Probability of CKD") +

theme_bw()

p_hist <- ggplot(nhs$variables, aes(x = Score_all)) +

geom_histogram(fill = "skyblue", color = "black", alpha = 0.7, bins = 30) +

labs(x = "LCD_score", y = "Frequency") +

theme_bw()

combined_plot <- p_main / p_hist + plot_layout(heights = c(3, 1))

print(combined_plot)

print("图形绘制完成！")

}

}

# 1. 加载必要的R包

library(rms) # 用于RCS分析、模型拟合和方差分析

library(ggplot2) # 用于高级图形绘制

library(patchwork) # 用于组合图形

# 2. 数据准备与rms包环境设置

# 假设您的数据框名为 'data'，且包含变量 'CKD' 和 'Score_all'

# 设置rms包所需的数据环境（关键步骤！）

ddist <- datadist(D1) # 将您的数据框名称替换为实际名称，例如 'data'

options(datadist = 'ddist')

# 3. 使用rms包的lrm函数拟合逻辑回归模型

# 注意：此处使用lrm函数（Logistic Regression Model），它专为二分类结局设计，并与rms包中的其他函数（如anova, Predict）完美兼容

model <- lrm(CKD ~ rcs(Score_all, 3), data = D1) # 使用3个节点，您可以根据AIC准则调整为4或5

# 4. 计算P for overall（总体关联性P值）和P for nonlinear（非线性P值）

# 使用rms包的anova函数进行方差分析，这是获取两个关键P值最直接的方法

model_anova <- anova(model)

print(model_anova) # 在控制台查看详细的方差分析表

# 从方差分析结果中提取P值

# 通常，输出表中对应您自变量的那一行，'Factor' 部分提供总体P值，'Nonlinear' 部分提供非线性P值

# 例如，如果您的自变量名为 'Score_all'，则：

# P_for_overall <- model_anova['Score_all', 'P'] # 总体P值

# P_for_nonlinear <- model_anova['Nonlinear', 'P'] # 非线性P值（在Score_all因子下）

# 更通用的方法是查看打印结果，手动记录或使用以下方式提取：

# 注意：anova输出的结构可能需要具体查看，以下提供一种参考方法

cat("P for overall (Score_all):", model_anova["Score_all", "P"], "\n")

cat("P for nonlinear:", model_anova["Nonlinear", "P"], "\n")

# 5. 使用Predict函数获取预测值及置信区间

# 生成一个覆盖Score_all整个数据范围的序列用于预测

pred_data <- data.frame(Score_all = seq(min(data$Score_all, na.rm = TRUE),

max(data$Score_all, na.rm = TRUE),

length.out = 100))

# 进行预测，得到每个Score_all值对应的OR值及其95%置信区间

pred_results <- Predict(model, Score_all, fun = exp, ref.zero = TRUE)

# fun=exp将log(Odds)转换为Odds Ratio (OR), ref.zero=TRUE将参考点设为OR=1处

# 6. 绘制图形

# (1) 绘制主图：RCS曲线与置信区间

p_main <- ggplot() +

geom_line(data = pred_results, aes(x = Score_all, y = yhat),

color = "red", linewidth = 1) + # 绘制OR值曲线

geom_ribbon(data = pred_results, aes(x = Score_all, ymin = lower, ymax = upper),

alpha = 0.3, fill = "red") + # 绘制置信区间

geom_hline(yintercept = 1, linetype = "dashed", color = "blue") + # 添加OR=1的参考线

labs(x = "LCD Score", y = "Odds Ratio (95% CI) for CKD",

title = "Dose-Response Relationship (RCS)",

subtitle = paste0("P for overall: ", round(model_anova["Score_all", "P"], 4),

"; P for nonlinear: ", round(model_anova["Nonlinear", "P"], 4))) +

theme_bw()

# (2) 绘制分布图：Score_all的直方图

p_hist <- ggplot(data, aes(x = Score_all)) +

geom_histogram(fill = "skyblue", color = "black", alpha = 0.7, bins = 30) +

labs(x = "LCD Score", y = "Frequency") +

theme_bw()

# (3) 组合图形：使用patchwork包将主图和分布图上下组合

combined_plot <- p_main / p_hist +

plot_layout(heights = c(3, 1)) # 主图高度占3份，分布图占1份

# 7. 显示最终图形

print(combined_plot)

#线性回归

library(survey)

library(car)

model <-svyglm(Score_all~ age + sex + ethic + BMI + alcohol.user2+

smoke2 +Hyperlipidemia+ Hypertension + HbA1c+ edu + poverty2+ DM_drug+acr+CKD_EPI_Scr_2009,nhs) |> reg_table(xlsx = 'x.xlsx',round = 3)

#————————————————————————————————————————————————————计算VIF

model <- svyglm(

Score_all ~ age + sex + ethic + BMI + alcohol.user2 + smoke2 +

Hyperlipidemia + Hypertension + HbA1c + edu + poverty2 + DM_drug + acr + CKD_EPI_Scr_2009,

design = nhs

)

# 2. 正确提取模型数据（关键步骤）

# ------------------------------------------

# 方法1：直接从设计对象中提取变量（推荐）

model_data <- nhs$variables[, all.vars(formula(model))]

# 3. 构建模型矩阵（处理因子变量）

X <- model.matrix(formula(model), data = model_data)

X <- X[, -1] # 去除截距项

# 4. 计算加权VIF（考虑调查权重）

# ------------------------------------------

# 提取权重向量

weights <- weights(nhs, "sampling")[complete.cases(model_data)] # 匹配完整数据

# 计算加权协方差矩阵

weighted_cor <- cov.wt(X, wt = weights, cor = TRUE)$cor

# 计算VIF（方差膨胀因子）

vif_values <- diag(solve(weighted_cor))

result <- data.frame(Variable = colnames(X), VIF = round(vif_values, 2))

# 输出结果

print(result[order(-result$VIF), ]) # 按VIF降序排列

#——————————————————————————————————————————————————————————————————————————————————————————————————————————

#4.2.2__________logstic

svy_uv.logit(subset(nhs, !(CKD_EPI_Scr_2009 <= 60 & acr <= 30) | CKD =='0'),y="CKD",

x=c('Score_all2', 'age' , 'sex' , 'ethic' , 'BMI2' ,'alcohol.user2',

'smoke2' ,'Hyperlipidemia', 'Hypertension' , 'HbA1c', 'edu' , 'poverty2', 'DM_drug'),round = 3)

svy_uv.logit(nhs,y="CKD",

x=c('Score_all3', 'age' , 'sex' , 'ethic' , 'BMI2' ,'alcohol.user2',

'smoke2' ,'Hyperlipidemia', 'Hypertension' , 'HbA1c', 'edu' , 'poverty2', 'DM_drug','energy5'),round = 3)

#_______________2)多因素logistic

f0 <- svyglm(CKD~Score_all3,nhs,family = quasibinomial)|> reg_table(xlsx = 'retinol0.xlsx',round = 3)

# write.xlsx(f0, 'E:/桌面/xxxx.xlsx')

f1 <- svyglm(CKD~Score_all3 + age + sex + ethic+BMI2,nhs,family = quasibinomial)|> reg_table(xlsx = 'retinol1.xlsx',round = 3)

# write.xlsx(f1, 'E:/桌面/xxxx.xlsx')

f2 <- svyglm(CKD~Score_all3 + age + sex + ethic +BMI2+ alcohol.user2+

smoke2+ edu + poverty2 + HbA1c+ DM_drug,nhs,

family = quasibinomial)|> reg_table(xlsx = 'retinol2.xlsx',round = 3)

# write.xlsx(f2, 'E:/桌面/xxxx.xlsx')

f3 <- svyglm(CKD~Score_all3 + age + sex + ethic + BMI2 + alcohol.user2+

smoke2 +Hyperlipidemia+ Hypertension + HbA1c+ edu + poverty2+ DM_drug, nhs,family = quasibinomial) |> reg_table(xlsx = 'retinol3.xlsx',round = 3)

# write.xlsx(f3, 'E:/桌面/xxxx.xlsx')

crude.Model.n(f0, f1, f2, f3,style = 1,round = 3,xlsx = 'retinol_cox')

f3 <- svyglm(CKD~Score_all3 + age + sex + ethic + BMI2 + alcohol.user2+

smoke2 +Hyperlipidemia+ Hypertension + HbA1c+ edu + poverty2+ DM_drug, nhs,family = quasibinomial)

#计算VIF————————————————————————————————————————————————————————————————————————————————————

# 安装并加载 jtools 包

if (!require(jtools)) install.packages("jtools")

library(jtools)

# 使用 summ 函数并设置 vif = TRUE 来获取 VIF

model_summary <- summ(f3, vif = TRUE, confint = FALSE)

# 从摘要中提取 VIF 值

vif_values_jtools <- model_summary$vif

print(vif_values_jtools)

#计算Pfor nonlinear————————————————————————————————————————————————————————————————————————————————

library(survey)

library(dplyr)

# 1. 在调查设计对象中创建LCDS的三分位数变量

# 首先计算LCDS评分的三分位点

tertile_breaks <- quantile(nhs$variables$Score_all,

probs = c(0, 1/3, 2/3, 1),

na.rm = TRUE)

# 创建三分位数分组变量

nhs$variables$LCDS_tertile <- cut(nhs$variables$Score_all,

breaks = tertile_breaks,

include.lowest = TRUE,

labels = c("T1", "T2", "T3")) # T1=最低三分位数组

# 检查分组情况

table(nhs$variables$LCDS_tertile, useNA = "ifany")

# 2. 拟合包含三分位数作为数值变量的模型

# 将三分位数转换为数值（1,2,3）以检验线性趋势

nhs$variables$LCDS_tertile_numeric <- as.numeric(nhs$variables$LCDS_tertile)

# 3. 在原有模型框架中加入数值型三分位数变量

# 替换原来的Score_all变量为数值型三分位数

f3_trend <- svyglm(CKD ~ LCDS_tertile_numeric + age + sex + ethic + BMI2 +

alcohol.user2 + smoke2 + Hyperlipidemia + Hypertension +

HbA1c + edu + poverty2 + DM_drug,

design = nhs, family = quasibinomial())

# 4. 提取线性趋势的P值（Wald检验）

model_summary <- summary(f3_trend)

p_trend <- model_summary$coefficients["LCDS_tertile_numeric", "Pr(>|t|)"]

# 显示结果

cat("=== 线性趋势检验结果 ===\n")

cat("P-value for linear trend across LCDS tertiles (Wald test):", round(p_trend, 4), "\n")

# 同时查看系数和OR值

cat("\n=== 详细模型输出 ===\n")

print(model_summary)

# 计算OR值及其95%置信区间

OR <- exp(coef(f3_trend)["LCDS_tertile_numeric"])

OR_CI <- exp(confint(f3_trend)["LCDS_tertile_numeric", ])

cat("\n=== 趋势强度估计 ===\n")

cat("OR per tertile increase:", round(OR, 3), "\n")

cat("95% CI: (", round(OR_CI[1], 3), ", ", round(OR_CI[2], 3), ")\n", sep = "")

f3 <- svyglm(energy3 + age + sex + ethic + BMI2 + alcohol.user2+

smoke2 +Hyperlipidemia+ Hypertension + HbA1c+ edu + poverty2+ DM_drug, nhs,family = quasibinomial) |> reg_table(xlsx = 'retinol3.xlsx',round = 3)

# write.xlsx(f3, 'E:/桌面/xxxx.xlsx')

f3 <- svyglm(CKD~Score_all3 + age + sex + ethic + BMI2 + alcohol.user2+

smoke2 +Hyperlipidemia+ Hypertension + HbA1c+ edu + poverty2+ DM_drug +energy3, subset(nhs,(CKD_EPI_Scr_2009 < 60 & acr > 30)),family = quasibinomial) |> reg_table(xlsx = 'retinol3.xlsx',round = 3)

f3 <- svyglm(CKD~Score_all3+ age + sex + ethic + BMI2 + alcohol.user2+

smoke2 +Hyperlipidemia+ Hypertension + HbA1c+ edu + poverty2+ DM_drug, subset(nhs, !(CKD_EPI_Scr_2009 <= 60 & acr <= 30) | CKD =='0'),family = quasibinomial) |> reg_table(xlsx = 'retinol3.xlsx',round = 3)

#_______________4)基线表

subset_data <- subset(nhs, (CKD_EPI_Scr_2009 < 60 & acr > 30))

# 检查样本量和CKD分布

nrow(subset_data)

table(subset_data$CKD)

# 检查CKD变量的类型和内容

str(nhs$CKD)

summary(nhs$CKD)

# 检查子集中CKD的缺失比例

sum(is.na(subset_data$CKD)) # 应该等于397（全部缺失）

#表1

f2 <- svy_tableone(design = nhs,cv = c('Score_all','age','HbA1c','acr','CKD_EPI_Scr_2009','energy'),c_meanSQse = T,

gv = c('sex','ethic','BMI2','alcohol.user2','smoke2','edu','poverty2','Hypertension','Hyperlipidemia',

'DM_drug','e_GFR2','acr2','RAS_Drug1','DM_drug','Year.x'),

g_perSQse = T,round = 3,by = 'Score_all3',total = T,xlsx = 'retinol_table2' )

f2 <- svy_tableone(design = nhs,cv = c('Score_all','age','HbA1c','acr','CKD_EPI_Scr_2009'),c_meanSQse = T,

gv = c('sex','ethic','BMI2','alcohol.user2','smoke2','edu','poverty2','Hypertension','Hyperlipidemia',

'DM_drug','e_GFR2','acr2','RAS_Drug1','DM_drug','Score_all3'),

g_perSQse = T,round = 3,by = 'Year.x',total = T,xlsx = 'retinol_table2' )

# 4.1.1 分层分析

##分层分析

stratum_model(object = nhs,

y='CKD',

x= 'Score_all3',stratum = 'age2',

adjust = c('sex','ethic','BMI2','alcohol.user2','smoke2','edu','poverty2','Hypertension','Hyperlipidemia',

'DM_drug','HbA1c'),

xlsx = "fc",round = 3,p = F)

stratum_model(object = nhs,

y='CKD',

x= 'sdLDL3',stratum = 'age2',

adjust = c('sex','ethic','BMI','alcohol.user2','smoke2'),

xlsx = "fc",round = 3,p = F)

stratum_model(object = nhs,

y='CKD',

x= 'Score_all3',stratum = 'sex',

adjust = c('age','ethic','BMI2','alcohol.user2','smoke2','edu','poverty2','Hypertension','Hyperlipidemia',

'DM_drug','HbA1c'),

xlsx = "fc",round = 3,p = F)

stratum_model(object = nhs,

y='CKD',

x= 'Score_all3',stratum = 'ethic',

adjust = c('age','sex','BMI2','alcohol.user2','smoke2','edu','poverty2','Hypertension','Hyperlipidemia',

'DM_drug','HbA1c'),

xlsx = "fc",round = 3,p = F)

stratum_model(object = nhs,

y='CKD',

x= 'Score_all3',stratum = 'Hyperlipidemia',

adjust = c('age','sex','BMI2','alcohol.user2','smoke2','edu','poverty2','Hypertension','ethic',

'DM_drug','HbA1c'),

xlsx = "fc",round = 3,p = F)

stratum_model(object = nhs,

y='CKD',

x= 'Score_all3',stratum = 'Hypertension',

adjust = c('age','sex','BMI2','alcohol.user2','smoke2','edu','poverty2','Hyperlipidemia','ethic',

'DM_drug','HbA1c'),

xlsx = "fc",round = 3,p = F)

stratum_model(object = nhs,

y='CKD',

x= 'Score_all3',stratum = 'HbA1c2',

adjust = c('age','sex','BMI2','alcohol.user2','smoke2','edu','poverty2','Hyperlipidemia','ethic','Hypertension',

'DM_drug'),

xlsx = "fc",round = 3,p = F)

stratum_model(object = nhs,

y='CKD',

x= 'Score_all3',stratum = 'BMI3',

adjust = c('age','sex','alcohol.user2','smoke2','edu','poverty2','Hyperlipidemia','ethic','Hypertension',

'DM_drug','HbA1c'),

xlsx = "fc",round = 3,p = F)

stratum_model(object = nhs,

y='CKD',

x= 'Score_all3',stratum = 'DM_drug',

adjust = c('age','sex','BMI','alcohol.user2','smoke2','edu','poverty2','Hyperlipidemia','ethic','Hypertension',

'HbA1c'),

xlsx = "fc",round = 3,p = F)

stratum_model(object = nhs,

y='CKD',

x= 'Score_all3',stratum = 'DM_drug',

adjust = c('age','sex','BMI','alcohol.user2','smoke2','edu','poverty2','Hyperlipidemia','ethic','Hypertension',

'HbA1c'),

xlsx = "fc",round = 3,p = F)

#___________________________________________________森林图

library(grid)

library(forestploter)

# 读取实例数据

dt <- LCD结果

# 筛选需要的列

dt <- dt[,1:6]

head(dt,6)

# 缩进

dt$Subgroup <- ifelse(is.na(dt$Retinol_intake),

dt$Subgroup,

paste0(" ", dt$Subgroup))

# NA 为空白或 NA 将转换为字符

dt$Retinol_intake <- ifelse(is.na(dt$Retinol_intake), "", dt$Retinol_intake)

# dt$Placebo <- ifelse(is.na(dt$Placebo), "", dt$Placebo)

dt$se <- (log(dt$hi) - log(dt$est))/1.96

# 为森林图添加空白列以显示 CI

dt$` ` <- paste(rep(" ", 20), collapse = " ")

# 创建置信区间列

dt$`HR (95% CI)` <- ifelse(is.na(dt$se), "",

sprintf("%.2f (%.2f to %.2f)",

dt$est, dt$low, dt$hi))

head(dt,100)

tm <- forest_theme(base_size = 10,

refline_col = "red",

footnote_col = "#636363",

footnote_fontface = "italic")

##参数设置

p <- forest(dt[,c(1:3, 8:9)],

est = dt$est,

lower = dt$low,

upper = dt$hi,

ci_column = 4,

ref_line = 1,

arrow_lab = c("Lower retinol intake", "Higher retinol intake"),

xlim = c(0, 1.5),

ticks_at = c(0, 0.5,1),

footnote = "This is the demo data. Please feel free to change\nanything you want.",

theme = tm)

plot(p)

#___________________________________________________森林图

library(grid)

library(forestploter)

# 读取实例数据

dt <- LCD结果

# 筛选需要的列

dt <- dt[,1:6]

# 缩进

dt$Subgroup <- ifelse(is.na(dt$Retinol_intake),

dt$Subgroup,

paste0(" ", dt$Subgroup))

# NA 为空白或 NA 将转换为字符

dt$Retinol_intake <- ifelse(is.na(dt$Retinol_intake), "", dt$Retinol_intake)

# dt$Placebo <- ifelse(is.na(dt$Placebo), "", dt$Placebo)

dt$se <- (log(dt$hi) - log(dt$est))/1.96

# 为森林图添加空白列以显示 CI

dt$` ` <- paste(rep(" ", 20), collapse = " ")

# 创建置信区间列

dt$`HR (95% CI)` <- ifelse(is.na(dt$se), "",

sprintf("%.2f (%.2f to %.2f)",

dt$est, dt$low, dt$hi))

head(dt,100)

tm <- forest_theme(base_size = 10,

refline_col = "red",

footnote_col = "#636363",

footnote_fontface = "italic")

##参数设置

p <- forest(dt[,c(1:3, 8:9)],

est = dt$est,

lower = dt$low,

upper = dt$hi,

ci_column = 4,

ref_line = 1,

arrow_lab = c("Lower LDH", "Higher LDH"),

xlim = c(0, 1.5),

ticks_at = c(0,0.5, 1),

footnote = "This is the demo data. Please feel free to change\nanything you want.",

theme = tm)

plot(p)
